# Cancer Trial Eligibility and Therapy Modifications for Individuals With Duffy Null–Associated Neutrophil Count

**DOI:** 10.1001/jamanetworkopen.2024.32475

**Published:** 2024-09-11

**Authors:** Stephen P. Hibbs, Laura Aiken, Kruti Vora, Chibuzo Mowete, Lauren E. Merz, Vanessa Apea, J. Mark Sloan, Christopher S. Lathan, Gregory A. Abel, Andrew Hantel

**Affiliations:** 1 Wolfson Institute of Population Health, Queen Mary University of London, London, United Kingdom; 2 Barts Health NHS Trust, London, United Kingdom; 3 Department of Medicine, Massachusetts General Hospital, Boston; 4 Blizard Institute, Queen Mary University of London, London, United Kingdom; 5 NHS Blood and Transplant, London, United Kingdom; 6 Department of Medical Oncology, Dana-Farber Cancer Institute, Boston, Massachusetts; 7 Center for Bioethics, Harvard Medical School, Boston, Massachusetts; 8 Department of Medicine, Boston University School of Medicine, Boston, Massachusetts

## Abstract

**Importance:**

Absolute neutrophil counts (ANCs) are used to determine cancer clinical trial (CCT) eligibility and systemic anticancer therapy (SACT) dose modifications. Duffy null–associated ANC (DANC) is a nonpathologic phenotype associated with lower ANC and most frequently seen in individuals with African and Middle Eastern ancestry. It is unclear whether CCTs exclude or SACT regimens modify doses for individuals with ANC within the DANC reference range.

**Objective:**

To investigate CCT exclusions and SACT dose modifications for ANC within the DANC reference range.

**Design, Setting, and Participants:**

This cross-sectional study of contemporary CCTs and SACT regimens included adult, interventional, phase 3 CCTs for the 5 most prevalent cancers in the US and United Kingdom (prostate, breast, melanoma, colorectal, and lung cancers) testing SACTs with start dates between November 1, 2021, and November 1, 2023, and that were registered on ClinicalTrials.gov. Preferred curative-intent SACT regimens were listed in National Comprehensive Cancer Network guidelines.

**Exposure:**

Cancer clinical trial exclusions and SACT regimen modifications.

**Main Outcome and Measures:**

Proportions of CCTs that exclude and SACT regimens that modify doses for individuals with an ANC within the DANC reference range.

**Results:**

For CCTs, 289 of 382 trials (75.7%) were eligible, of which 221 (76.5% [95% CI, 71.1%-81.2%]) excluded patients with ANC values within the DANC reference range. Colorectal CCTs had the highest (38 of 44 [86.4% (95% CI, 72.6%-94.8%)]) and prostate CCTs had the lowest (11 of 23 [47.8% (95% CI, 26.8%-69.4%)]) proportions of exclusions. Of CCTs testing cytotoxic chemotherapy, 116 of 142 (81.7% [95% CI, 74.3%-87.7%]) had exclusions; 93 of 123 (75.6% [95% CI, 67.0%-82.9%]) CCTs testing targeted therapies alone and 12 of 24 (50.0% [95% CI, 29.1%-70.9%]) testing hormonal therapies alone had exclusions. Among the 116 US- and UK-based trials, exclusions were present in 78 (67.2% [95% CI, 57.9%-75.7%]). Of 71 SACT regimens, 38 (53.5% [95% CI, 41.3%-65.5%]) included dose modifications for ANC values within the DANC reference range. Lung cancer regimens had the highest (23 of 31 [74.2% (95% CI, 55.4%-88.1%)]) and prostate cancer had the lowest (0 of 12 [0 (95% CI, 0%-26.4%)]) proportions of modifications. Regimens including chemotherapy had modifications in 32 of 44 (72.7% [95% CI, 57.2%-85.0%]); 11 of 20 (55.0% [95% CI, 31.5%-76.9%]) of targeted therapy regimens and 0 of 16 (0% [95% CI, 0%-20.6%]) of hormonal therapy regimens had modifications. Among regimens including chemotherapy and/or targeted therapy, modifications were present in 38 of 55 (69.1% [95% CI, 49.7%-73.2%]).

**Conclusions and Relevance:**

In this cross-sectional study, substantial proportions of CCTs excluded and SACT regimens modified doses for patients with ANCs in the DANC reference range. These practices structurally discriminate against patients of African and Middle Eastern ancestry. While determining optimal SACT dose modifications requires further study, CCT exclusion criteria should be revised.

## Key Points

**Question:**

Do cancer clinical trials and anticancer regimens restrict participation of individuals with Duffy null–associated absolute neutrophil counts (DANCs) by setting exclusions and dose modifications within their neutrophil reference range?

**Findings:**

In this cross-sectional analysis of 289 phase 3 trials and 71 curative-intent anticancer regimens for the 5 most prevalent US and UK cancers, 77% of trials and 54% of regimens excluded or modified doses for patients with neutrophil counts within the DANC reference range.

**Meaning:**

These findings suggest that eligibility criteria and dose modifications structurally inhibit individuals with DANC from participating in trials and obtaining appropriate anticancer therapy.

## Teaser

This cross-sectional study assesses whether phase 3 cancer clinical trials and systemic anticancer therapy regimens continue to exclude or recommend dose modifications for absolute neutrophil counts among individuals with the Duffy null–associated absolute neutrophil count phenotype.

## Introduction

Disparities in survival for persons with cancer from racially and ethnically minoritized groups are persistent and not fully accounted for by socioeconomic and disease-related factors.^1,2^ A central cause of persistent survival disparities is structural racism, which “systematically fosters discrimination through mutually enforcing systems,”^1^ including barriers to participating in cancer clinical trials (CCT) or obtaining appropriate systemic anticancer therapy (SACT).^3,4^ An unaddressed factor that may promote both disparate CCT exclusion and suboptimal SACT delivery for individuals with African or Middle Eastern ancestry is the Duffy null–associated absolute neutrophil count (DANC). The Duffy null phenotype is associated with 40% lower circulating ANCs than individuals with the Duffy nonnull phenotype due to preferential localization of the neutrophils to the spleen and is not associated with an increased risk of infection.^5,6^ Driven by selective pressure from *Plasmodium vivax* infection, 80% to 100% of individuals from West Africa and two-thirds of self-identified African American individuals have the Duffy null phenotype, of whom 10% to 17% have an ANC less than 1500/μL at baseline (to convert to ×10^9^/L, multiply by 0.001).^7,8^

The limited data available suggest that neutrophil-related criteria might unnecessarily exclude individuals with DANC from CCT participation.^9,10^ Neutrophil criteria may also mandate unnecessary SACT delays, dose reductions, or discontinuations (collectively referred to as modifications).^11^ This is particularly concerning, as SACT dose intensity strongly correlates with survival,^12^ such as in breast cancer, where Black women have inferior survival, and lower dose intensity is associated with lower pretreatment ANCs.^13,14,15^ The contemporary use of ANC criteria that exclude individuals with DANC from CCTs or limit them from receiving appropriate dose intensity remains unclear. We hypothesized that phase 3 CCTs and SACT regimens for prevalent cancers continue to exclude or recommend dose modifications for individuals with ANC values within the DANC reference range.

## Methods

### Study Design, Objectives, and ANC Definition

This study included 2 cross-sectional analyses. The objective of the first (CCT eligibility) was to identify the proportion of phase 3 CCTs that would exclude individuals from participating for ANC values within the DANC reference range. The objective of the second (SACT dose modification) was to identify the proportion of regimens with recommended dose modifications for ANC values within the DANC reference range. The reference range lower limit for DANC was defined as 1200/μL.^5^ This study followed the Strengthening the Reporting of Observational Studies in Epidemiology (STROBE) reporting guidelines. As this study analyzed data from clinical trial protocols and FDA labels, it was not subject to institutional review board review per US Health and Human Services requirements.^16^

### CCT Eligibility Analysis

This cohort consisted of adult, interventional, phase 3 trials of the 5 most prevalent cancers in the US and UK (breast cancer, prostate cancer, lung cancer [including small cell and non–small cell], colorectal cancer, and cutaneous melanoma)^17,18^ with start dates between November 1, 2021, and November 1, 2023. ClinicalTrials.gov was selected as the data source because of its status as a regulatory requirement and international use. The database was queried on November 3, 2023. A data extraction template was generated based on prior studies^19^; the cohort was independently screened and extracted by 2 individuals (S.P.H. and L.A.). Discrepancies were resolved by consensus (interrater reliability κ = 0.86). Additional search and extraction procedures are described in the eMethods and eTable 1 in Supplement 1. Exclusion criteria related to ANC were categorized as (1) explicit, defined as a quantitative exclusion of individuals for ANC values within the DANC reference range (eg, <1500/uL); (2) implicit, defined as a qualitative exclusion of individuals for ANC values within the DANC reference range (eg, “abnormal laboratory values” or requiring “adequate hematologic function”); and (3) none, defined as no criteria that would exclude individuals for ANC values within the DANC reference range.

### SACT Dose Modification Analysis

There is no single data source analogous to ClinicalTrials.gov from which to identify recommended SACT regimens and ANC-related dose modifications; therefore, American Society of Clinical Oncology recommendations for establishing safe chemotherapy administration practices were followed to identify regimens and recommended dose modifications for the same cancers analyzed in the CCT eligibility cohort.^20,21^ As the balance between dose intensity and toxicity is most in favor of dose intensity in the curative setting, this cohort was restricted to curative-intent regimens. First, we identified current evidence-based guidelines from the National Comprehensive Cancer Network (NCCN). Second, guidelines were reviewed to identify recommended SACT regimens. Third, recommended dose modifications were identified from the protocols and/or manuscripts of the trial(s) cited. When modifications could not be identified, an evidence-based medical decision support tool (UpToDate; UpToDate, Inc) was reviewed and recommendations were cataloged. Regimens were categorized as explicitly, implicitly, or not dose modifying. Definitions of explicit and implicit were analogous to the CCT eligibility analysis. In case of discrepancies between cited trials, the less restrictive categorization was retained. The NCCN guideline versions were current as of February 11, 2024; interrater reliability was κ = 0.77. To assess the robustness of this analysis, the US Food and Drug Administration (FDA) labels for the individual SACT agents that comprised NCCN-identified regimens were also identified and analyzed separately to determine recommended dose modifications. Additional search and extractions methods are detailed in the eMethods and eTable 2 in Supplement 1.

### Statistical Analysis

Results were reported using descriptive statistics with frequencies and percentages with 95% CIs using the binomial exact method. Crude values were the sum of the total trials or regimens in each exclusion or modification category across cancer types. As the number of trials and regimens varied by cancer type, weighted values were generated by calculating exclusion or modification category proportions within each cancer type, dividing those proportions by the number of cancer types in the calculation, and summing the categories. Statistical testing was performed using STATA, version 16.1 (StataCorp LLC). Interrater reliability was calculated using the Cohen κ for the CCT cohort (study eligibility and type of SACT and category of exclusion criteria) and for the SACT dose modification analysis (regimen eligibility and category of modification criteria); no weighting was used.

## Results

### CCT Eligibility

**Figure 1.**
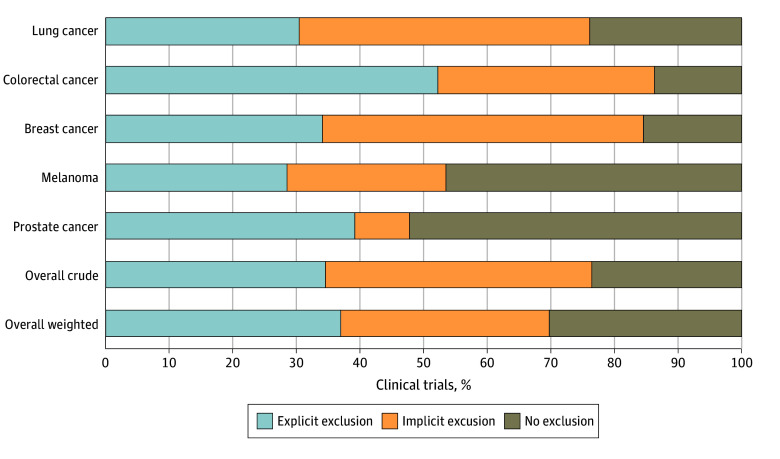
The Proportions of Cancer Clinical Trials That Excluded Patients for Absolute Neutrophil Counts (ANCs) Within the Duffy Null–Associated ANC Reference Range by Cancer Type and Type of Restriction Bars representing individual cancer types show all trials including each cancer type (prostate [n = 23], melanoma [n = 28], breast [n = 97], colorectal [n = 44], and lung [n = 105]). Overall crude values are for unique trials (n = 289), and overall weighted values weight each cancer type equally and include duplicates across cancer types (n = 297).

The search resulted in 382 unique phase 3 CCTs, of which 289 (75.7%) were eligible and constituted the analytic cohort (eTable 3 in Supplement 1). eFigure 1 in Supplement 1 shows search results and exclusions by cancer type. Within the analytic cohort, 221 trials (76.5% [95% CI, 71.1%-81.2%]) explicitly or implicitly excluded patients for ANC values within the DANC reference range. Exclusions by type of cancer and restriction are shown in Figure 1 and by type of therapy in eFigure 3 in Supplement 1. Colorectal CCTs had the highest (38 of 44 [86.4% (95% CI, 72.6%-94.8%)]) and prostate CCTs had the lowest (11 of 23 [47.8% (95% CI, 26.8%-69.4%)]) proportions of exclusions. Of trials that included cytotoxic chemotherapy, 116 of 142 (81.7% [95% CI, 74.3%-87.7%]) had exclusions; 93 of 123 (75.6% [95% CI, 67.0%-82.9%]) testing targeted therapy without chemotherapy and 12 of 24 (50.0% [95% CI, 29.1%-70.9%]) testing hormonal therapy only had exclusions. When restricted to US- and UK-based trials (n = 116), exclusions were present in 78 (67.2% [95% CI, 57.9%-75.7%]) (eFigure 4 in Supplement 1).

### SACT Dose Modifications

**Figure 2.**
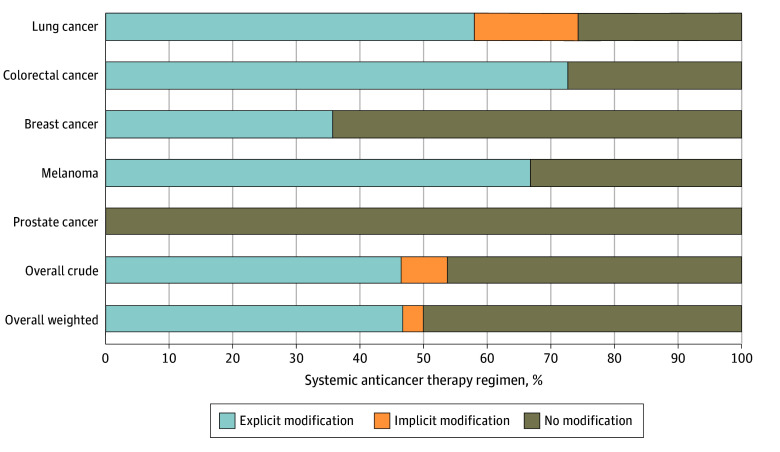
The Proportions of Systemic Anticancer Therapy Regimen Dose Modifications That Excluded Patients for Absolute Neutrophil Counts (ANCs) Within the Duffy Null–Associated ANC Reference Range by Cancer Type and Type of Modification Bars representing individual cancer types are mutually exclusive (lung [n = 31], colorectal [n = 11], breast [n = 14], melanoma [n = 3], and prostate [n = 12]) and summed for the overall crude result. The overall weighted result weights each cancer type equally prior to summing.

There were 71 curative-intent SACT regimens in the analysis consisting of 50 individual SACT agents (eTable 4 in Supplement 1). Of the 71 regimens, 38 (53.5% [95% CI, 41.3%-65.5%]) recommended dose modifications for ANC values within the DANC reference range. Dose modifications by type of cancer and modification are shown in Figure 2 and by type of therapy in eFigure 5 in Supplement 1. Lung cancer had the highest proportion of regimens with modifications (23 of 31 [74.2% (95% CI, 55.4%-88.1%)]) and prostate cancer had the lowest (0 of 12 [0% (95% CI, 0%-26.4%)]). Regimens including chemotherapy had modifications in 32 of 44 (72.7% [95% CI, 57.2%-85.0%]), while 11 of 20 (55.0% [95% CI, 31.5%-76.9%]) of those including targeted therapy and 0 of 16 (0% [95% CI, 0%-20.6%]) including hormonal therapy had modifications. Excluding hormone-only regimens, modifications were present in 38 of 55 (69.1% [95% CI, 49.7%-73.2%]) (eFigure 6 in Supplement 1). By FDA labels, 44 of 71 (62.0% [95% CI, 49.7%-73.2%]) of regimens had modifications (eFigure 7 in Supplement 1).

## Discussion

In these cross-sectional analyses, substantial proportions of CCTs and SACT regimens unnecessarily excluded individuals with DANC from trial participation and recommended treatment intensity reductions for ANC values in the DANC reference range. This is an example of structural racism that impacts those with African or Middle Eastern ancestry and is highly amenable to remediation. In the US, DANC and Gilbert syndrome—which impacts bilirubin levels and predominately affects White individuals—are both present in approximately 10% of the population.^22,23^ Unlike DANC, Gilbert-specific eligibility criteria and dose modifications are long-standing and widespread.

For CCTs, eligibility criteria can and should be immediately revised to include a DANC-specific ANC threshold. Given the 40% difference in ANC between individuals with Duffy null and Duffy nonnull phenotypes and the common ANC criterion of at least 1500/μL, a reasonable DANC-specific eligibility threshold would be 900/μL. At minimum, the DANC-specific criterion should be below the lower limit of the DANC reference range (1200/μL). Dose modifications to SACT require additional study to identify the optimal balance between dose intensity and neutropenia-related toxicity. One reasonable approach would be comparative effectiveness testing of standard of care regimens using DANC-specific and DANC-agnostic dose modifications. Through this, rates of avoided dose modifications and neutropenia-related complications—and their collective impact on response and outcomes—could be determined.

### Limitations

This study has limitations, including the dose modification identification methods, which followed guidelines but cannot universally reflect practice, and that regimen use is not homogenous but regimens were analyzed with equal weight. This assessment was also limited to trials registered on ClinicalTrials.gov. Due to statutory registration requirements, there is excellent coverage of US- and UK-based trials but potentially incomplete coverage of trials from countries that predominantly use other registries; this may limit generalizability to those countries. The NCCN guidelines and FDA labels are also most applicable to the US, although these data sources and the trials underlying them are commonly used in other countries for determining dose modifications. For the CCT cohort, while explicit exclusion criteria are unambiguous, implicit criteria may not exclude individuals with DANCs if investigators are aware of this issue and DANC-specific reference ranges are available. This is unlikely to have occurred during the study period, however, as reference ranges were only recently published and a national effort to promote DANC-specific research and clinical practices has just begun.^24^

## Conclusions

The findings of this study indicate that current eligibility criteria and dose modifications discriminate against individuals with DANCs; their remediation is likely to impact disparities in trial enrollment and cancer outcomes. While determining optimal SACT dose modifications for individuals with DANCs requires further study, CCT exclusion criteria for individuals with DANCs should be revised.

## Notes

Supplement 1. **eMethods.** Search, Screening, and Data Extraction Procedures **eTable 1.** CCT Cohort Data Extraction Template **eTable 2.** SACT Cohort Data Extraction Template **eTable 3.** CCT Cohort **eTable 4.** SACT Regimen Cohort **eFigure 1.** CCT Cohort Search Results and Exclusions **eFigure 2.** SACT Cohort Search Results and Exclusions **eFigure 3.** Proportions of Clinical Trials That Exclude Patients for ANC Values Within the DANC Reference Range by Type of Therapy and Type of Restriction **eFigure 4.** Proportions of Clinical Trials Within the US and UK That Exclude Patients for ANC Values Within the DANC Reference Range by Cancer Type and Type of Restriction **eFigure 5.** Proportions of SACT Regimens With Dose Modifications That Exclude Patients for ANC Values Within the DANC Reference Range, by Type of Therapy and Type of Restriction **eFigure 6.** Chemotherapy and Targeted Therapy Dose Modifications That Exclude Patients for ANC Values Within the DANC Reference Range, by Cancer Type and Type of Restriction **eFigure 7.** FDA Label-Based SACT Dose Modifications That Exclude Patients for ANC Values Within the DANC Reference Range, by Cancer Type and Type of Restriction
eReferences.

Supplement 2.
Data Sharing Statement
