# Error in Abstract, Results, and Supplement 1

**DOI:** 10.1001/jamanetworkopen.2024.56980

**Published:** 2024-12-19

In the Original Investigation titled “Cancer Trial Eligibility and Therapy Modifications for Individuals With Duffy Null–Associated Neutrophil Count,”^1^ published September 11, 2024, errors occurred in statements concerning the sensitivity analysis in the Results portions of the Abstract and article body as well as in data presented in eFigures 1 and 4 in Supplement 1. An inadvertent transposition error in the study dataset had miscategorized the US and UK status of the trials. This article has been corrected.^1^
