# Supplementary material for: Cancer Trial Eligibility and Therapy Modifications for Individuals With Duffy Null–Associated Neutrophil Count
Source: JAMA Netw Open. 2024 Sep 11;7(9):e2432475. doi: 10.1001/jamanetworkopen.2024.32475 (PMC11391325; doi:10.1001/jamanetworkopen.2024.32475)
Supplement: Supplement 1. — eMethods. Search, Screening, and Data Extraction Procedures eTable 1. CCT Cohort Data Extraction Template eTable 2. SACT Cohort Data Extraction Template eTable 3. CCT Cohort eTable 4. SACT Regimen Cohort eFigure 1. CCT Cohort Search Results and Exclusions eFigure 2. SACT Cohort Search Results and Exclusions eFigure 3. Proportions of Clinical Trials That Exclude Patients for ANC Values Within the DANC Reference Range by Type of Therapy and Type of Restriction eFigure 4. Proportions of Clinical Trials Within the US and UK That Exclude Patients for ANC Values Within the DANC Reference Range by Cancer Type and Type of Restriction eFigure 5. Proportions of SACT Regimens With Dose Modifications That Exclude Patients for ANC Values Within the DANC Reference Range, by Type of Therapy and Type of Restriction eFigure 6. Chemotherapy and Targeted Therapy Dose Modifications That Exclude Patients for ANC Values Within the DANC Reference Range, by Cancer Type and Type of Restriction eFigure 7. FDA Label-Based SACT Dose Modifications That Exclude Patients for ANC Values Within the DANC Reference Range, by Cancer Type and Type of Restriction eReferences. [file jamanetwopen-e2432475-s001.pdf]

Supplementary Online Content

Hibbs SP, Aiken L, Vora K, et al. Cancer trial eligibility and therapy modifications for individuals with Duffy null–associated neutrophil count. *JAMA Netw Open*. 2024;7(9):e2432475. doi:10.1001/jamanetworkopen.2024.32475

eMethods. Search, Screening, and Data Extraction Procedures

eTable 1. CCT Cohort Data Extraction Template

eTable 2. SACT Cohort Data Extraction Template

eTable 3. CCT Cohort

eTable 4. SACT Regimen Cohort

eFigure 1. CCT Cohort Search Results and Exclusions

eFigure 2. SACT Cohort Search Results and Exclusions

eFigure 3. Proportions of Clinical Trials That Exclude Patients for ANC Values Within the DANC Reference Range by Type of Therapy and Type of Restriction

eFigure 4. Proportions of Clinical Trials Within the US and UK That Exclude Patients for ANC Values Within the DANC Reference Range by Cancer Type and Type of Restriction

eFigure 5. Proportions of SACT Regimens With Dose Modifications That Exclude Patients for ANC Values Within the DANC Reference Range, by Type of Therapy and Type of Restriction

eFigure 6. Chemotherapy and Targeted Therapy Dose Modifications That Exclude Patients for ANC Values Within the DANC Reference Range, by Cancer Type and Type of Restriction

eFigure 7. FDA Label-Based SACT Dose Modifications That Exclude Patients for ANC Values Within the DANC Reference Range, by Cancer Type and Type of Restriction

eReferences.

This supplementary material has been provided by the authors to give readers additional information about their work.

© 2024 Hibbs SP et al. *JAMA Network Open*.

eMethods. Search, Screening, and Data Extraction Procedures

ClinicalTrials.gov

Database selection: clinicaltrials.gov was selected as the database of choice because US and UK regulations mandate registration and publication of US clinical trial records on this site, it captures a large number of global clinical trials, and it supplies adequate data fields for selecting trials of interest. As UK regulations allow trials to be alternatively registered on ISRCTN, we assessed the coverage gap of using clinicaltrials.gov alone by performing a search of cancer clinical trials registered on ISRCTN and reviewing a random selection of 20 trial records that fit the search criteria below to assess for cross-registration on clinicaltrials.gov. As 100% of this selection was cross-registered, the search and data extraction were limited to clinicaltrials.gov.

Search: The following searches were performed using the categories and options available on clinicaltrials.gov. If a category or option is not mentioned, it was not included in the search. Separate searches were performed for each condition/disease term listed. Of note, the searches were not restricted to trials based in the United States or United Kingdom, but a sensitivity analysis of those trials was performed. All searches were performed on 3 November 2023.

- Condition/Disease: (each performed as separate search)
    - colorectal cancer
    - breast cancer
    - prostate cancer
    - melanoma
    - lung cancer
- Study Status: All studies
- Study Phase: III
- Study Type: Interventional
- Study Start:
    - From: 1 November 2021
    - To: 1 November 2023

Screening: Search results were manually screened to exclude trials that did not test SACT (including but not limited to cytotoxic chemotherapeutics, small molecule inhibitors, antibodies, immunomodulators, hormonal therapies, and injected radioisotopes). We excluded trials that were non-interventional, diagnostic only, or tested non-SACT interventions only (such as radiotherapy, surgical, or supportive therapy trials). Studies were grouped into the following mutually exclusive categories based on the type of SACT used.

a. Chemotherapy: included cytotoxic chemotherapeutics, with or without Targeted Therapy or Hormonal Therapy.
b. Targeted Therapy: included targeted therapeutics (e.g., small molecule inhibitors, antibodies, immunomodulators) but not Chemotherapy, with or without Hormonal Therapy.

© 2024 Hibbs SP et al. *JAMA Network Open*.

c. Hormonal Therapy: included hormonal therapeutics but not Chemotherapy or Targeted Therapy.

Data Extraction: For studies included for further review, corresponding clinicaltrials.gov records were reviewed, and eligibility criteria extracted verbatim. If a full trial protocol was attached to the registry entry, the trial protocol was reviewed. Criteria were categorized into the mutually exclusive categories as outlined in the methods with verbatim criteria extracted for any difficult categorizations. Each study was screened and extracted independently by two reviewers. Discrepancies were resolved by consensus, with a third reviewer adjudicating if needed. Studies identified in more than one search (e.g., recruited patients with breast cancer and colorectal cancer) were retained in each search; in reporting of overall statistics the study was counted once, and in reporting for the individual cancer types the study was counted under each applicable cancer type.

© 2024 Hibbs SP et al. *JAMA Network Open*.

SACT regimen and FDA label search, screening, and data extraction procedures.

The following methods adhered, as applicable, to American Society for Clinical Oncology (ASCO) guidance on the development of standardized SACT dose modifications for a clinical oncology practice from these published articles and their references: *2016 Updated American Society of Clinical Oncology/Oncology Nursing Society Chemotherapy Administration Safety Standards, Including Standards for Pediatric Oncology* and *Principles of Safe Practice Using an Oncology EHR System for Chemotherapy Ordering, Preparation, and Administration, Part 2 of 2*.[1,2]

Of note, Food and Drug Administration (FDA) label search and analysis was performed separately as the regimen-based modifications were thought to account for emergent toxicities from combination therapy that may not be addressed in individual agent labels.

Search:
SACT Regimens: NCCN Clinical Practice Guidelines in Oncology Evidence Blocks for breast cancer, colorectal cancer, non-small cell lung cancer, small cell lung cancer, prostate cancer, and cutaneous melanoma were obtained on 11 February 2024. Two study team members independently reviewed the guidelines and identified and verbatim catalogued "Preferred" SACT regimens used during curative treatment and the citations supporting the use of the regimen. After screening, the clinical trial manuscripts referenced were identified through PubMed and available study protocols were obtained. UpToDate was searched between 20 February and 6 March 2024 to identify the regimen's treatment protocol (e.g., "Treatment Protocols for Non-small Cell Lung Cancer," available at uptodate.com/contents/treatment-protocols-for-non-small-cell-lung-cancer).

FDA Labels: After regimen screening was completed, each SACT agent was catalogued. Drugs@FDA (www.accessdata.fda.gov/scripts/cder/daf/index.cfm) was queried between 25 February and 3 March 2024 with the agent's name, and the most recent New Drug Application label was reviewed. If no New Drug Application label was available, the most recent Abbreviated New Drug Application label was reviewed.

Screening:
SACT Regimens: Each reviewer screened the regimens that were identified and removed regimens for the following reasons: duplicate regimens, regimens that could not be confirmed as Preferred, and regimens that were not independent treatments (i.e., maintenance therapy only given after another SACT regimen). Screening results are shown in eFigure 2.

FDA Labels: There was no screening of FDA labels beyond ensuring the correct agent and New Drug Application label was being reviewed.

Data Extraction:
SACT Regimens: Protocols were the preferred source of recommended dose modifications. When a protocol was available, each reviewer reviewed the latest version of the protocol and dose modifications were catalogued verbatim. When a protocol was not available, the

© 2024 Hibbs SP et al. *JAMA Network Open*.

manuscript was reviewed, and dose modifications catalogued if available. When neither had dose modification information, UpToDate was reviewed, and the dose modification listed was catalogued. Regimens were independently categorized into the mutually exclusive categories of *explicitly modifying*, *implicitly modifying*, or *not modifying*, using analogous criteria to the CCT Eligibility analysis. Each citation for a given regimen was reviewed, and if there were discrepancies between dose modification categories between trials referenced, the less restrictive categorization was retained. Discrepancies were resolved by consensus, with a third reviewer adjudicating if needed.

FDA Labels: Each SACT agent used in a regimen was reviewed and dose modification information was catalogued verbatim and categorized as outlined above. Information from each agent was then assessed as a regimen, and the most restrictive categorization was retained. Discrepancies were resolved by consensus, with a third reviewer adjudicating if needed.

© 2024 Hibbs SP et al. *JAMA Network Open*.

eTable 1. CCT cohort data extraction template.

Source of data element	Data element	Description of data element	Notes
Clinicaltrials.gov	NCT Number	Clinical trial registration number	
	Study Title	Title of Study	
	Study URL	Link to clinicaltrials.gov study record	
	Brief Summary	Description of trial	
	Conditions	Condition/disease treated	
	Interventions	Drugs, procedures, devices, or biological agents being tested	
	Sponsor	Name of trial sponsor	
	Phases	Trial phase (II/III or III)	
	Funder Type	Primary trial funder: NIH, Other Government, Industry, Network, Other	
	Study Type	Check to ensure study is listed as interventional	
	Locations	Sites at which study is being performed	
Study team	Cancer Type	Categorization of cancer type into broad disease categories: breast, lung, prostate, melanoma, colorectal	
	Systemic Anti-Cancer Therapy (SACT) Tested	Yes/No	These rows were aligned across reviewers; discrepancies were then catalogued and reviewed in a consensus conference to make a final determination.
	Type of SACT Tested	None, Chemotherapy Only, Hormonal Therapy Only, Targeted Therapy Only, Combination	
	Harmonized Include/Exclude Decision	Include/Exclude	These rows report the final determination made from the consensus conference above.
	Harmonized Type of SACT Tested	None, Chemotherapy Only, Hormonal Therapy Only, Targeted Therapy Only, Combination	
	Full protocol available	Yes/No	
	Any neutrophil-related criteria present	Yes/No	These rows were aligned across reviewers; discrepancies were then catalogued and reviewed in a consensus conference to make a final determination
	Explicit neutrophil count-based criteria present	Yes/No	
	Free text comments on neutrophil-related criteria	Describe neutrophil criteria	

© 2024 Hibbs SP et al. *JAMA Network Open*.

	Harmonized neutrophil category	No restriction, implicit restriction, explicit restriction	These rows report the final determination made from the consensus conference above.
	Additional details on harmonized neutrophil category	Any additional neutrophil criteria description needed	

© 2024 Hibbs SP et al. *JAMA Network Open*.

eTable 2. SACT cohort data extraction template.

Source of data element	Data element	Description of data element	Notes
NCCN guidelines	NCCN Regimen	Systemic anticancer therapy regimens as listed on NCCN	Treatment schema and Evidence Block listings were used to identify regimens. Regimens were listed verbatim.
Entered by study team	Cancer Type	Categorization of cancer type into broad disease categories: breast, lung, prostate, melanoma, colorectal	
	Type of SACT Tested	None, Chemotherapy Only, Hormonal Therapy Only, Targeted Therapy Only, [Combinations]	This row was aligned across reviewers; discrepancies were then catalogued and reviewed in a consensus conference to make a final determination.
	Trial(s) referenced in NCCN guidelines	Link to trial(s) with primary data for the NCCN regimen listed	
	Full protocol available	Yes/No	
	Free text comments on protocol selection	Description of whether protocol or manuscript details dose modification criteria	
	UpToDate protocol link	Link to UpToDate page/table for NCCN regimen if dose modification details not found in trial or protocol	
	Category of neutrophil count-based dose modification criteria	No ANC-related changes required, quantitative restriction within healthy ANC range, quantitative restriction below healthy ANC range, qualitative restriction of ANC or quantitative restriction of WBC/leukocyte count, Unclear – see free text	These rows were aligned across reviewers; discrepancies were then catalogued and reviewed in a consensus conference to make a final determination
	Free text comments on neutrophil-related dose modification criteria	Description of neutrophil count-related dose modification criteria as listed in therapy regimen protocols or UpToDate	
	Harmonized neutrophil category	No restriction, explicit restriction, implicit restriction	This row reports the final determination made from the consensus conference above.
FDA labels	SACT agent name	Name of individual agent	
	Type of SACT agent	Chemotherapy, Hormonal Therapy, Targeted Therapy	

© 2024 Hibbs SP et al. *JAMA Network Open*.

	FDA label link	URL of FDA label	
	Category of neutrophil count-based dose modification criteria	No ANC-related changes required, quantitative restriction within healthy ANC range, quantitative restriction below healthy ANC range, qualitative restriction of ANC or quantitative restriction of WBC/leukocyte count, Unclear – see free text	These rows were aligned across reviewers; discrepancies were then catalogued and reviewed in a consensus conference to make a final determination
	Free text comments on neutrophil-related dose modification criteria	Description of neutrophil count-related dose modification criteria as listed in label	
	Harmonized neutrophil category	No restriction, explicit restriction, implicit restriction	This row reports the final determination made from the consensus conference above.

© 2024 Hibbs SP et al. *JAMA Network Open*.

eTable 3. CCT Cohort. Duplicate trials were removed, and the first instance of the trial is retained here.

Cancer Type	NCT Number	Title	URL
Breast	NCT06057610	A Phase III Study of SHR-A1811 Injection With or Without Pertuzumab in HER2-Positive Recurrent or Metastatic Breast Cancer	https://clinicaltrials.gov/study/NCT06057610
Breast	NCT05860465	Safety and Efficacy of SPH4336 in Combination With Endocrine Therapy in the Treatment of Locally Advanced or Metastatic Breast Cancer	https://clinicaltrials.gov/study/NCT05860465
Breast	NCT05954442	Everolimus With Investigator's Choice of Chemotherapy in Advanced Triple-Negative Breast Cancer (TNBC) With Luminal Androgen Receptor (LAR) Subtype	https://clinicaltrials.gov/study/NCT05954442
Breast	NCT05753865	A Study to Evaluate the Efficacy and Safety in Patients With Advanced Breast Cancer Treated With SYHX2011 Compared to Paclitaxel for Injection (Albumin-bound)	https://clinicaltrials.gov/study/NCT05753865
Breast	NCT05253066	Chidamide Combined With Exemestane (+/- Goserelin) Versus Neoadjuvant Chemotherapy in Patients of Stage II-III HR-positive/HER2-negative Breast Cancer	https://clinicaltrials.gov/study/NCT05253066
Breast	NCT05501886	Gedatolisib Plus Fulvestrant With or Without Palbociclib vs Standard-of-Care for the Treatment of Patients With Advanced or Metastatic HR+/HER2- Breast Cancer (VIKTORIA-1)	https://clinicaltrials.gov/study/NCT05501886
Breast	NCT05929768	Shorter Chemo-Immunotherapy Without Anthracycline Drugs for Early-Stage Triple Negative Breast Cancer	https://clinicaltrials.gov/study/NCT05929768
Breast	NCT05415215	A Study to Evaluate Patient Preference for Home Administration of Fixed-Dose Combination of Pertuzumab and Trastuzumab for Subcutaneous Administration in Participants With Early or Locally Advanced/Inflammatory HER2-Positive Breast Cancer	https://clinicaltrials.gov/study/NCT05415215
Breast	NCT05114720	Moxifloxacin in Adjuvant Treatment of Patients With Operable Breast Cancer	https://clinicaltrials.gov/study/NCT05114720
Breast	NCT05950945	Trastuzumab Deruxtecan (T-DXd) in Patients Who Have Hormone Receptor-negative and Hormone Receptor-positive HER2-low or HER2 IHC 0 Metastatic Breast Cancer	https://clinicaltrials.gov/study/NCT05950945
Breast	NCT05232916	Phase 3 Study to Evaluate the Efficacy and Safety of HER2/Neu Peptide GLSI-100 (GP2 + GM-CSF) in HER2/Neu Positive Subjects	https://clinicaltrials.gov/study/NCT05232916
Breast	NCT05883852	EC-THP Versus TCbHP in HER2-positive Lymph Node Positive Early Breast Cancer	https://clinicaltrials.gov/study/NCT05883852
Breast	NCT05696626	Evaluation of Lasofoxifene Combined With Abemaciclib Compared With Fulvestrant Combined With Abemaciclib in Locally Advanced or Metastatic ER+/HER2- Breast Cancer With an ESR1 Mutation	https://clinicaltrials.gov/study/NCT05696626
Breast	NCT05720026	Study to Evaluate the Efficacy and Safety of SYSA1901 vs. Perjeta¬Æ of HER2-Positive Breast Cancer	https://clinicaltrials.gov/study/NCT05720026

© 2024 Hibbs SP et al. *JAMA Network Open*.

Breast	NCT05909397	A Study of ARV-471 (PF-07850327) Plus Palbociclib Versus Letrozole Plus Palbociclib in Participants With Estrogen Receptor Positive, Human Epidermal Growth Factor Negative Advanced Breast Cancer	https://clinicaltrials.gov/study/NCT05909397
Breast	NCT05812807	Pembrolizumab vs. Observation in People With Triple-negative Breast Cancer Who Had a Pathologic Complete Response After Chemotherapy Plus Pembrolizumab	https://clinicaltrials.gov/study/NCT05812807
Breast	NCT05169567	Abemaciclib (LY2835219) Plus Fulvestrant Compared to Placebo Plus Fulvestrant in Previously Treated Breast Cancer	https://clinicaltrials.gov/study/NCT05169567
Breast	NCT05189067	Efficacy and Safety of Adjuvant Docetaxel and Trastuzumab in Stage I HER2-positive Breast Cancer	https://clinicaltrials.gov/study/NCT05189067
Breast	NCT05814354	SHR-A1811 Versus Investigator's Chemotherapy in Recurrent/Metastatic Breast Cancer Clinical Trial	https://clinicaltrials.gov/study/NCT05814354
Breast	NCT05207514	Compare the Efficacy and the Safety of Doxorubicin and Cyclophosphamide Followed by Taxotere Versus Doxorubicin and Cyclophosphamide Nanoxel M as Neoadjuvant Chemotherapy in Breast Cancer	https://clinicaltrials.gov/study/NCT05207514
Breast	NCT05851014	A Study of GB491 in Combination With Letrozole in Patients With HR Positive and HER2 Negative Advanced Breast Cancer	https://clinicaltrials.gov/study/NCT05851014
Breast	NCT05838066	Efficacy and Safety of KN026 in Combination With HB1801 in the First-line Treatment of Subjects With HER2-positive Recurrent or Metastatic Breast Cancer.	https://clinicaltrials.gov/study/NCT05838066
Breast	NCT05861830	Dalpiciclib With Endocrine Therapy for Advanced Breast Cancer After CDK4/6 Inhibitor Failure (DAWNA-FES)	https://clinicaltrials.gov/study/NCT05861830
Breast	NCT05744687	Phase II/III Study of SPH4336 Combined With Letrozole vs Placebo Combined With Letrozole in First-line Treatment of Breast Cancer	https://clinicaltrials.gov/study/NCT05744687
Breast	NCT05374512	A Study of Dato-DXd Versus Investigator's Choice Chemotherapy in Patients With Locally Recurrent Inoperable or Metastatic Triple-negative Breast Cancer, Who Are Not Candidates for PD-1/PD-L1 Inhibitor Therapy (TROPION-Breast02)	https://clinicaltrials.gov/study/NCT05374512
Breast	NCT05755048	FS-1502 Versus T-DM1 for HER2-Positive Unresectable Locally Advanced or Metastatic Breast Cancer	https://clinicaltrials.gov/study/NCT05755048
Breast	NCT05774951	A Study of Camizestrant in ER+/HER2- Early Breast Cancer After at Least 2 Years of Standard Adjuvant Endocrine Therapy	https://clinicaltrials.gov/study/NCT05774951
Breast	NCT05999149	A Study of Camrelizumab Plus Chemotherapy in Combination With or Without Famitinib as Neoadjuvant Therapy in Participants With Triple Negative Breast Cancer (TNBC)	https://clinicaltrials.gov/study/NCT05999149
Breast	NCT05077449	A Study of XZP-3287 in Combination With Fulvestrant in Patients With Advanced Breast Cancer	https://clinicaltrials.gov/study/NCT05077449

© 2024 Hibbs SP et al. *JAMA Network Open*.

Breast	NCT05207709	Ribociclib vs. Palbociclib in Patients With Advanced Breast Cancer Within the HER2-Enriched Intrinsic Subtype	https://clinicaltrials.gov/study/NCT05207709
Breast	NCT05555706	Study of B013 and Nab-Paclitaxel for Locally Advanced or Metastatic Triple Negative Breast Cancer	https://clinicaltrials.gov/study/NCT05555706
Breast	NCT06009627	Study of Neoadjuvant Endocrine Therapy in HR Positive and HER2 Negative Premenopausal Breast Cancer Patients	https://clinicaltrials.gov/study/NCT06009627
Breast	NCT05891093	Efficacy and Safety of Fluzoparib Combined With Adjuvant Endocrine Therapy for HR+/HER2- SNF3-subtype Early Breast Cancer	https://clinicaltrials.gov/study/NCT05891093
Breast	NCT05894239	A Study to Evaluate the Efficacy and Safety of Inavolisib in Combination With Phesgo Versus Placebo in Combination With Phesgo in Participants With PIK3CA-Mutated HER2-Positive Locally Advanced or Metastatic Breast Cancer	https://clinicaltrials.gov/study/NCT05894239
Breast	NCT05889871	A Randomized, Controlled, Open-label Clinical Trial of Adjuvant Intensive Therapy for HR+/ HER2-SNF4 Early Breast Cancer	https://clinicaltrials.gov/study/NCT05889871
Breast	NCT05879926	Evaluating the Addition of Adjuvant Chemotherapy to Ovarian Function Suppression Plus Endocrine Therapy in Premenopausal Patients With pN0-1, ER-Positive/HER2-Negative Breast Cancer and an Oncotype Recurrence Score Less Than or Equal to 25	https://clinicaltrials.gov/study/NCT05879926
Breast	NCT05132582	A Study of Tucatinib or Placebo With Trastuzumab and Pertuzumab for Metastatic HER2+ Breast Cancer	https://clinicaltrials.gov/study/NCT05132582
Breast	NCT05159193	Neoadjuvant Treatment Pegylated Liposomal Doxorubicin Plus Cyclophosphamide Sequential Docetaxel Plus Trastuzumab and Pertuzumab Versus Docetaxel Plus Carboplatin Combined With Trastuzumab and Pertuzumab in HER-2 Positive Breast Cancer	https://clinicaltrials.gov/study/NCT05159193
Breast	NCT05212454	Efficacy of Supplement Adjuvant Capecitabine in HR+/HER2- Breast Cancer Patients With High Risks	https://clinicaltrials.gov/study/NCT05212454
Breast	NCT05633654	Study of Sacituzumab Govitecan-hziy and Pembrolizumab Versus Treatment of Physician's Choice in Patients With Triple Negative Breast Cancer Who Have Residual Invasive Disease After Surgery and Neoadjuvant Therapy	https://clinicaltrials.gov/study/NCT05633654
Breast	NCT05474690	A Study Comparing the Efficacy of TCbHP and ECHP-THP in the Neoadjuvant Treatment of HER2-positive Breast Cancer	https://clinicaltrials.gov/study/NCT05474690
Breast	NCT05514054	A Study of Imlunestrant Versus Standard Endocrine Therapy in Participants With Early Breast Cancer	https://clinicaltrials.gov/study/NCT05514054
Breast	NCT05670054	Cyclin Dependant Kinase 4/6 (CDK4/6) Inhibitors as a Second Line Treatment in Metastatic Breast Cancer Patients	https://clinicaltrials.gov/study/NCT05670054
Breast	NCT05552001	Safety and Efficacy Analysis of an Antibody Associated With a Chemotherapy for Patients With a Triple Negative Metastatic Breast Cancer	https://clinicaltrials.gov/study/NCT05552001

© 2024 Hibbs SP et al. *JAMA Network Open*.

Breast	NCT05646862	A Study Evaluating the Efficacy and Safety of Inavolisib Plus Fulvestrant Compared With Alpelisib Plus Fulvestrant in Participants With HR-Positive, HER2-Negative, PIK3CA Mutated, Locally Advanced or Metastatic Breast Cancer Post CDK4/6i and Endocrine Combination Therapy	https://clinicaltrials.gov/study/NCT05646862
Breast	NCT05172518	Utidelone Plus Capecitabine Versus Taxane Plus Capecitabine in HER2-negative Locally Advanced or Metastatic Breast Cancer	https://clinicaltrials.gov/study/NCT05172518
Breast	NCT05747794	Study in Metastatic Breast Cancer Patients Receiving Eftilagimod Alpha or Placebo in Combination With Paclitaxel Chemotherapy	https://clinicaltrials.gov/study/NCT05747794
Breast	NCT05901428	TCb vs EC-T in High Risk ER+/HER2- Breast Cancer	https://clinicaltrials.gov/study/NCT05901428
Breast	NCT06044623	Implementing Geriatric Assessment for Dose Optimization of Cyclin-dependent Kinase (CDK) 4/6-inhibitors in Older Breast Cancer Patients	https://clinicaltrials.gov/study/NCT06044623
Breast	NCT05347134	SKB264 Injection vs Investigator Selected Regimens to Treat Locally Advanced, Recurrent or Metastatic Triple-negative Breast Cancer	https://clinicaltrials.gov/study/NCT05347134
Breast	NCT05122494	A Phase ,Ö¢ Study of the Efficacy and Safety of Hemay022+Aromatase Inhibitor(AI) in Participants With ER+/HER2+ Advanced or Metastatic Breast Cancer	https://clinicaltrials.gov/study/NCT05122494
Breast	NCT05654623	A Study to Learn About a New Medicine Called ARV-471 (PF-07850327) in People Who Have Advanced Metastatic Breast Cancer.	https://clinicaltrials.gov/study/NCT05654623
Breast	NCT05760378	Famitinib in Combination With Camrelizumab and TPC in The First-line Treatment of Immunomodulatory Locally Advanced or Metastatic TNBC.	https://clinicaltrials.gov/study/NCT05760378
Breast	NCT05439499	This is a Multicenter, Randomized, Double-blind, Placebo-controlled Phase III Clinical Study Evaluating the Efficacy and Safety of FCN-437c Versus Placebo in Combination With Letrozole or Anastrozole ¬± Goserelin in Women With HR+ and HER2- Advanced Breast Cancer.	https://clinicaltrials.gov/study/NCT05439499
Breast	NCT05806060	Precise Treatment for BLIS Subtype of TNBC in the First-line Treatment of Locally Advanced or Metastatic Breast Cancer	https://clinicaltrials.gov/study/NCT05806060
Breast	NCT05375461	TQB3616 Capsules Plus Fulvestrant Compared to Placebo Plus Fulvestrant in Previously Treated Breast Cancer in Clinical Trail	https://clinicaltrials.gov/study/NCT05375461
Breast	NCT05288777	Adjuvant Chemoradiation and Biomarkers of Response in High-risk Breast Cancer	https://clinicaltrials.gov/study/NCT05288777
Breast	NCT05871918	A Multicenter, Randomized, Open, Phase III Trial of ddEC-THPvs Evaluating the Efficacy and Safety of TCHP Neoadjuvant Therapy for HER2-positive Breast Cancer	https://clinicaltrials.gov/study/NCT05871918
Breast	NCT05909332	Study of Antivascular Therapy Combined With Chemotherapy Versus Chemotherapy in Adjuvant Therapy of TNBC-BLIS Patients.	https://clinicaltrials.gov/study/NCT05909332
Breast	NCT05128773	Study of Amcenestrant (SAR439859) Versus Tamoxifen for Patients With Hormone Receptor-positive (HR+) Early Breast Cancer, Who Have Discontinued Adjuvant Aromatase Inhibitor Therapy Due to Treatment-related Toxicity	https://clinicaltrials.gov/study/NCT05128773

© 2024 Hibbs SP et al. *JAMA Network Open*.

Breast	NCT05862064	PD-1 Combined With Adjuvant Chemotherapy and Antivascular Therapy Versus Chemotherapy Alone in Patients With Operable Triple-negative Breast Cancer	https://clinicaltrials.gov/study/NCT05862064
Breast	NCT05512364	TREAT ctDNA Elacestrant	https://clinicaltrials.gov/study/NCT05512364
Breast	NCT05438810	This is a Multicenter, Randomized, Double-blind, Placebo-controlled Phase III Clinical Study Evaluating the Efficacy and Safety of FCN-437c in Combination With Fluvestrant ¬± Goseraline Versus Placebo Combined With Fulvestrant ¬± Goserelin in Women With HR+ and HER2- Advanced Breast Cancer.	https://clinicaltrials.gov/study/NCT05438810
Breast	NCT05283837	A Study to Evaluate Safety, Efficacy, Pharmacokinetics, and Immunogenicity of Test Pertuzumab (ZRC-3277, Cadila Healthcare Ltd.,)	https://clinicaltrials.gov/study/NCT05283837
Breast	NCT05078047	Study Comparing the Standard Administration of IO Versus the Same IO Administered Each 3 Months in Patients With Metastatic Cancer in Response After 6 Months of Standard IO	https://clinicaltrials.gov/study/NCT05078047
Breast	NCT06018337	A Study of DB-1303 vs Investigator's Choice Chemotherapy in Metastatic Breast Cancer	https://clinicaltrials.gov/study/NCT06018337
Breast	NCT05780567	Clinical Study on Adjuvant Therapy of TQB3616 Combined With Endocrine Therapy Compared With Placebo Combined With Endocrine Therapy in Patients With Breast Cancer	https://clinicaltrials.gov/study/NCT05780567
Breast	NCT05382286	Study of Sacituzumab Govitecan-hziy and Pembrolizumab Versus Treatment of Physician's Choice and Pembrolizumab in Patients With Previously Untreated, Locally Advanced Inoperable or Metastatic Triple-Negative Breast Cancer	https://clinicaltrials.gov/study/NCT05382286
Breast	NCT05698186	Thero2-01S22 in HER2-positive Breast Cancer	https://clinicaltrials.gov/study/NCT05698186
Breast	NCT05388500	Protocol for Herceptin as Adjuvant Therapy With Reduced Exposure to Chemotherapy (PHARE-C)	https://clinicaltrials.gov/study/NCT05388500
Breast	NCT05629585	A Study of Dato-DXd With or Without Durvalumab Versus Investigator's Choice of Therapy in Patients With Stage I-III Triple-negative Breast Cancer Without Pathological Complete Response Following Neoadjuvant Therapy (TROPION-Breast03)	https://clinicaltrials.gov/study/NCT05629585
Breast	NCT05426486	A Study of ARX788 Combined With Pyrotinib Maleate Versus TCBHP (Trastuzumab Plus Pertuzumab With Docetaxel and Carboplatin) as Neoadjuvant Treatment in HER2-positive Breast Cancer Patients	https://clinicaltrials.gov/study/NCT05426486
Breast	NCT05365178	To Evaluate the Efficacy and Safety of TQB3616 in Combination With Flulvesant Versus Placebo in Combination With Flulvesant in Previously Untreated Hormone-receptor (HR)-Positive, Human Epidermal Growth Factor Receptor 2 (HER2)-Negative Advanced Breast Cancer	https://clinicaltrials.gov/study/NCT05365178
Breast	NCT06072612	Study of the Bria-IMT Regimen and CPI vs Physicians' Choice in Advanced Metastatic Breast Cancer.	https://clinicaltrials.gov/study/NCT06072612

© 2024 Hibbs SP et al. *JAMA Network Open*.

Breast	NCT05760612	A Clinical Study on Hormone Receptor Positive HER2 Positive Breast Cancer of RCB1-2 After Neoadjuvant Treatment With Trastuzumab Combined With Parezumab	https://clinicaltrials.gov/study/NCT05760612
Breast	NCT05673629	Utidelone in Combination With AC Versus Docetaxel in Combination With AC for Neoadjuvant Chemotherapy in Patients With HER2-negative Breast Cancer	https://clinicaltrials.gov/study/NCT05673629
Breast	NCT06016738	OP-1250 (Palazestrant) vs. Standard of Care for the Treatment of ER+/HER2- Advanced Breast Cancer	https://clinicaltrials.gov/study/NCT06016738
Breast	NCT05802225	Clinical Study of the Efficacy and Safety of BCD-178 and Perjeta¬Æ as Neoadjuvant Therapy of HER2-Positive Breast Cancer	https://clinicaltrials.gov/study/NCT05802225
Breast	NCT05257395	A Study of XZP-3287 in Combination With Letrozole/Anastrozole in Patients With Advanced Breast Cancer	https://clinicaltrials.gov/study/NCT05257395
Breast	NCT05841381	Adjuvant Study of Pyrotinib in Combination With Trastuzumab in HER2 Positive Invasive Breast Cancer	https://clinicaltrials.gov/study/NCT05841381
Breast	NCT05904964	Disitamab Vedotin (RC48) in Hormone Receptor Positive, HER2-low Metastatic Breast Cancer (the Rosy Trial)	https://clinicaltrials.gov/study/NCT05904964
Breast	NCT06058377	Adding an Immunotherapy Drug, MEDI4736 (Durvalumab), to the Usual Chemotherapy Treatment (Paclitaxel, Cyclophosphamide, and Doxorubicin) for Stage II-III Breast Cancer	https://clinicaltrials.gov/study/NCT06058377
Breast	NCT05910398	Continuous or Intermittent Extension of Adjuvant Pyrotinib for Invasive HER2-positive Breast Cancer	https://clinicaltrials.gov/study/NCT05910398
Breast	NCT05952557	An Adjuvant Endocrine-based Therapy Study of Camizestrant (AZD9833) in ER+/HER2- Early Breast Cancer (CAMBRIA-2)	https://clinicaltrials.gov/study/NCT05952557
Breast	NCT05296798	A Study to Evaluate the Efficacy and Safety of Giredestrant in Combination With Phesgo (Pertuzumab, Trastuzumab, and Hyaluronidase-zzxf) Versus Phesgo in Participants With Locally Advanced or Metastatic Breast Cancer (heredERA Breast Cancer)	https://clinicaltrials.gov/study/NCT05296798
Breast	NCT05306340	A Study Evaluating the Efficacy and Safety of Giredestrant Plus Everolimus Compared With The Physician's Choice of Endocrine Therapy Plus Everolimus in Participants With Estrogen Receptor-Positive, HER2-Negative, Locally Advanced or Metastatic Breast Cancer (evERA Breast Cancer)	https://clinicaltrials.gov/study/NCT05306340
Breast	NCT05065411	Efficacy & Safety Evaluation of Enobosarm in Combo With Abemaciclib in Treatment of ER+HER2- Metastatic Breast Cancer	https://clinicaltrials.gov/study/NCT05065411
Breast	NCT05840211	Study of Sacituzumab Govitecan Versus Treatment of Physician's Choice in Patients With Hormone Receptor-positive/Human Epidermal Growth Factor Receptor 2 Negative (HR+/HER2-) Metastatic Breast Cancer Who Have Received Endocrine Therapy	https://clinicaltrials.gov/study/NCT05840211

© 2024 Hibbs SP et al. *JAMA Network Open*.

Breast	NCT05430399	Utidelone Versus Docetaxel in HER2-negative Locally Advanced or Metastatic Breast Cancer	https://clinicaltrials.gov/study/NCT05430399
Breast	NCT05346224	A Study to Evaluate the Efficacy and Safety of HLX11 vs. EU-Perjeta¬Æ in the Neoadjuvant Therapy of HER2-Positive and HR-Negative Early-stage or Locally Advanced Breast Cancer	https://clinicaltrials.gov/study/NCT05346224
Breast	NCT05433480	A Study of BPI-16350 in Combination With Fulvestrant in Patients With HR+ and HER2- Locally Advanced, Recurrent or Metastatic Breast Cancer	https://clinicaltrials.gov/study/NCT05433480
Breast	NCT05645536	Safety Extension Study for Subjects With HR+, HER2- Breast Cancer for Subjects Who Have Completed the OVELIA Study	https://clinicaltrials.gov/study/NCT05645536
Breast	NCT05134194	A Study of Camrelizumab in Combination With Chemotherapy Regimen Comparative Chemotherapy Regimen for Metastatic Triple-negative Breast Cancer	https://clinicaltrials.gov/study/NCT05134194
Breast	NCT05382299	Study of Sacituzumab Govitecan-hziy Versus Treatment of Physician's Choice in Patients With Previously Untreated Metastatic Triple-Negative Breast Cancer	https://clinicaltrials.gov/study/NCT05382299
Breast	NCT05424835	A Trial of SHR-A1811versus Pyrotinib in Combination With Capecitabine in HER2-Positive, Unresectable and/or Metastatic Breast Cancer Subjects Previously Treated With Trastuzumab and Taxane	https://clinicaltrials.gov/study/NCT05424835
Breast	NCT05901935	DP303c in Patients With HER2-positive Advanced Breast Cancer	https://clinicaltrials.gov/study/NCT05901935
Breast	NCT05038735	Study to Assess the Efficacy and Safety of Alpelisib Plus Fulvestrant in Participants With HR-postitive (HR+), HER2-negative, Advanced Breast Cancer After Treatment With a CDK4/6 Inhibitor and an Aromatase Inhibitor.	https://clinicaltrials.gov/study/NCT05038735
Prostate	NCT05116475	Evaluation of dAroLutamide Addition to anDrogen Deprivation Therapy and radIatioN Therapy in Newly Diagnosed Prostate Cancer With Pelvic Lymph Nodes Metastases	https://clinicaltrials.gov/study/NCT05116475
Prostate	NCT05786716	DETERMINE Trial Treatment Arm 04: Trastuzumab in Combination With Pertuzumab in Adult, Teenage/Young Adult and Paediatric Patients With Cancers With HER2 Amplification or Activating Mutations	https://clinicaltrials.gov/study/NCT05786716
Prostate	NCT05627752	Docetaxel Alone or in Combination With Enzalutamide for mCRPC Previously Treated With Abiraterone at mHSPC Stage	https://clinicaltrials.gov/study/NCT05627752
Prostate	NCT05169112	Impact of Hormonal Therapy on Prostate Cancer Recurrence After Radical Prostatectomy	https://clinicaltrials.gov/study/NCT05169112
Prostate	NCT05288166	A Study of Abemaciclib (LY2835219) With Abiraterone in Men With Prostate Cancer That Has Spread to Other Parts of the Body and is Expected to Respond to Hormonal Treatment (Metastatic Hormone-Sensitive Prostate Cancer)	https://clinicaltrials.gov/study/NCT05288166
Prostate	NCT05348577	Study of Capivasertib + Docetaxel vs Placebo + Docetaxel as Treatment for Metastatic Castration Resistant Prostate Cancer (mCRPC)	https://clinicaltrials.gov/study/NCT05348577
Prostate	NCT05204927	177Lu-PSMA-I&T for Metastatic Castration-Resistant Prostate Cancer	https://clinicaltrials.gov/study/NCT05204927

© 2024 Hibbs SP et al. *JAMA Network Open*.

Prostate	NCT05794906	A Study to Compare Darolutamide Given With Androgen Deprivation Therapy (ADT) With ADT in Men With Hormone Sensitive Prostate Cancer and Raise of Prostate Specific Antigen (PSA) Levels After Local Therapies	https://clinicaltrials.gov/study/NCT05794906
Prostate	NCT05590793	Effects of Triptorelin Pamoate 6-month When Given to Adult Chinese Participants With Advanced Cancer in the Prostate	https://clinicaltrials.gov/study/NCT05590793
Prostate	NCT05781217	Short Versus Long-term Androgen Deprivation Therapy With Salvage Radiotherapy in Prostate Cancer. URONCOR 0624	https://clinicaltrials.gov/study/NCT05781217
Prostate	NCT05956639	Comparing a 6-month vs Long-term Course of Rezvilutamide With ADT Plus Chemotherapy in mHSPC	https://clinicaltrials.gov/study/NCT05956639
Prostate	NCT05676203	A Trial Comparing Docetaxel 75 mg/m2 (3w) Versus Docetaxel 50 mg/m2 (2w) in Combination With Darolutamide + ADT in mHSPC Patients	https://clinicaltrials.gov/study/NCT05676203
Prostate	NCT05009290	A Trial of SHR3680 in Prostate Cancer Patients Who Are Candidates for Radical Prostatectomy	https://clinicaltrials.gov/study/NCT05009290
Prostate	NCT05458856	Effects of Triptorelin When Given Every 6-months Under the Skin to Adult Males With Cancer in the Prostate	https://clinicaltrials.gov/study/NCT05458856
Prostate	NCT05352178	Metastasis-directed Therapy for Oligorecurrent Prostate Cancer	https://clinicaltrials.gov/study/NCT05352178
Prostate	NCT05050084	Two Studies for Patients With Unfavorable Intermediate Risk Prostate Cancer Testing Less Intense Treatment for Patients With a Low Gene Risk Score and Testing a More Intense Treatment for Patients With a Higher Gene Risk Score	https://clinicaltrials.gov/study/NCT05050084
Prostate	NCT05605964	Randomized Study to Evaluate MACE in Patients With Prostate Cancer Treated With Relugolix or Leuprolide Acetate	https://clinicaltrials.gov/study/NCT05605964
Prostate	NCT05983783	Comparing the Efficacy and Safety of Rezvilutamide+ADT+Docetaxel Versus Rezvilutamide +ADT in the mHSPC	https://clinicaltrials.gov/study/NCT05983783
Prostate	NCT05191680	TherApeutics in Early ProState Cancer (TAPS02)	https://clinicaltrials.gov/study/NCT05191680
Prostate	NCT05884398	A Study of an Intermittent ADT Approach With Apalutamide Monotherapy in Participants With mCSPC	https://clinicaltrials.gov/study/NCT05884398
Prostate	NCT04916613	ADT +/- Darolutamide in de Novo Metastatic Prostate Cancer Patients With Vulnerable Functional Ability (PEACE6-Vulnerable)	https://clinicaltrials.gov/study/NCT04916613
Prostate	NCT05771896	Darolutamide With Radium-223 or Placebo and the Effect on Radiological Progression-Free Survival for Patients With mCSPC	https://clinicaltrials.gov/study/NCT05771896
Prostate	NCT03348670	Pharmacogenomics IND EXEMPT SNP Clinical Study - Abiraterone and Single Nucleotide Polymorphisms	https://clinicaltrials.gov/study/NCT03348670
Lung	NCT05671510	ONC-392 Versus Docetaxel in Metastatic NSCLC That Progressed on PD-1/PD-L1 Inhibitors	https://clinicaltrials.gov/study/NCT05671510
Lung	NCT05989542	A Confirmatory Clinical Study in NSCLC Patients With MET Exon 14 Mutation (KUNPENG-2)	https://clinicaltrials.gov/study/NCT05989542

© 2024 Hibbs SP et al. *JAMA Network Open*.

Lung	NCT05668988	A Study of DZD9008 Versus Platinum-Based Doublet Chemotherapy in Local Advanced or Metastatic Non-small Cell Lung Cancer	https://clinicaltrials.gov/study/NCT05668988
Lung	NCT05058651	Evaluating the Addition of the Immunotherapy Drug Atezolizumab to Standard Chemotherapy Treatment for Advanced or Metastatic Neuroendocrine Carcinomas That Originate Outside the Lung	https://clinicaltrials.gov/study/NCT05058651
Lung	NCT05756972	A Study of PM8002 (Anti-PD-L1/VEGF) in Combination With Chemotherapy in Patients With NSCLC	https://clinicaltrials.gov/study/NCT05756972
Lung	NCT05116462	Neoadjuvant and Adjuvant Therapy Studies of Sindilizumab in Resectable Lung Cancer	https://clinicaltrials.gov/study/NCT05116462
Lung	NCT05687266	Study of Datopotamab Deruxtecan (Dato-DXd) in Combination With Durvalumab and Carboplatin for First-Line Treatment of Patients With Advanced NSCLC Without Actionable Genomic Alterations	https://clinicaltrials.gov/study/NCT05687266
Lung	NCT05496166	The Efficiency of Surgery and Radiotherapy After SHR-1316 (Adebrelimab) and Platinum-containing Doublet Induction Therapy for Limited-stage Small Cell Lung Cancer	https://clinicaltrials.gov/study/NCT05496166
Lung	NCT05740566	Study Comparing Tarlatamab With Standard of Care Chemotherapy in Relapsed Small Cell Lung Cancer	https://clinicaltrials.gov/study/NCT05740566
Lung	NCT05042375	A Trial of Camrelizumab Combined With Famitinib Malate in Treatment Na√Øve Subjects With PD-L1-Positive Recurrent or Metastatic Non-Small Cell Lung Cancer	https://clinicaltrials.gov/study/NCT05042375
Lung	NCT05668650	Double-blind Study to Evaluate the PK, Efficacy, Safety and Immunogenicity of MB12 Versus Keytruda¬Æ in Stage IV NSCLC	https://clinicaltrials.gov/study/NCT05668650
Lung	NCT05609968	Study of Pembrolizumab (MK-3475) Monotherapy Versus Sacituzumab Govitecan in Combination With Pembrolizumab for Participants With Metastatic Non-small Cell Lung Cancer (NSCLC) With Programmed Cell Death Ligand 1 (PD-L1) Tumor Proportion Score (TPS) ‚â•50% (MK-3475-D46)	https://clinicaltrials.gov/study/NCT05609968
Lung	NCT05844150	A Study of PM8002 (Anti-PD-L1/VEGF) in Combination With Chemotherapy in Patients With ES-SCLC	https://clinicaltrials.gov/study/NCT05844150
Lung	NCT05800015	A Trial to Learn How the Combination of Fianlimab With Cemiplimab and Chemotherapy Works Compared With Cemiplimab and Chemotherapy for Treating Adult Patients With Advanced Non-small Cell Lung Cancer	https://clinicaltrials.gov/study/NCT05800015
Lung	NCT05722015	A Study of Subcutaneous (SC) Pembrolizumab Coformulated With Hyaluronidase (MK-3475A) vs Intravenous Pembrolizumab in Adult Participants With Metastatic Non-small Cell Lung Cancer (NSCLC) (MK-3475A-D77)	https://clinicaltrials.gov/study/NCT05722015
Lung	NCT05690945	A Study of QL1706 in Combination With Chemotherapy in PD-L1-Negative Non-small Cell Lung Cancer	https://clinicaltrials.gov/study/NCT05690945
Lung	NCT06020352	The Efficacy and Safety of KN046 Combined With Axitinib	https://clinicaltrials.gov/study/NCT06020352

© 2024 Hibbs SP et al. *JAMA Network Open*.

Lung	NCT06031597	Radiotherapy Combined With ICIs as Treatment for LA-NSCLC After Failing Induction Immunochemotherapy	https://clinicaltrials.gov/study/NCT06031597
Lung	NCT05785767	A Study to Learn if a Combination of Fianlimab and Cemiplimab Versus Cemiplimab Alone is More Effective for Adult Participants With Advanced Non-Small Cell Lung Cancer (NSCLC)	https://clinicaltrials.gov/study/NCT05785767
Lung	NCT05111197	Local Ablative Stereotactic Radiotherapy for Residual Hypermetabolic Lesion in Patients With Locally Advanced or Metastatic Non-small Cell Lung Cancer Long-term Responders to Immunotherapy	https://clinicaltrials.gov/study/NCT05111197
Lung	NCT05132075	Study of JDQ443 in Comparison With Docetaxel in Participants With Locally Advanced or Metastatic KRAS G12C Mutant Non-small Cell Lung Cancer	https://clinicaltrials.gov/study/NCT05132075
Lung	NCT05209256	Alflutinib Versus Alflutinib Plus Chemotherapy for NSCLC	https://clinicaltrials.gov/study/NCT05209256
Lung	NCT05654454	A Safety and Efficacy Study of Bevacizumab, Paclitaxel, Carboplatin Compared to Avastin¬Æ in Non-Small Cell Lung Cancer	https://clinicaltrials.gov/study/NCT05654454
Lung	NCT05502237	Zimberelimab and Domvanalimab in Combination With Chemotherapy Versus Pembrolizumab With Chemotherapy in Patients With Untreated Metastatic Non-Small Cell Lung Cancer	https://clinicaltrials.gov/study/NCT05502237
Lung	NCT05091567	A Phase III, Open-Label Study of Maintenance Lurbinectedin in Combination With Atezolizumab Compared With Atezolizumab in Participants With Extensive-Stage Small-Cell Lung Cancer	https://clinicaltrials.gov/study/NCT05091567
Lung	NCT05791097	Study of Efficacy and Safety of Ociperlimab in Combination With Tislelizumab and Platinum-based Doublet Chemotherapy as First-line Treatment for Participants With Locally Advanced or Metastatic NSCLC.	https://clinicaltrials.gov/study/NCT05791097
Lung	NCT05346952	A Study of TQB2450 Injection Plus Chemotherapy Followed by TQB2450 Plus Anlotinib Versus Tislelizumab Plus Chemotherapy Followed by Tislelizumab in the Treatment of First-line Non-squamous Non-small Cell Lung Cancer(NSCLC).	https://clinicaltrials.gov/study/NCT05346952
Lung	NCT05922345	Evaluation of the Efficacy and Safety of AL2846 Capsule Combined With TQB2450 Injection Compared to Docetaxel Injection in Advanced Non-small Cell Lung Cancer Patients Who Have Failed With Immunotherapy.	https://clinicaltrials.gov/study/NCT05922345
Lung	NCT05725343	A Prevention Trial of Canakinumab in Subjects at High Risk for Lung Cancer	https://clinicaltrials.gov/study/NCT05725343
Lung	NCT06048315	A Single Center, Single Arm Clinical Study on the Treatment of Advanced Non-small Cell Lung Cancer With Positive EGFR Sensitive Mutations and Failed EGFR TKIs With the Combination of Enrotinib and Paclitaxel Monoclonal Antibody	https://clinicaltrials.gov/study/NCT06048315
Lung	NCT05450692	A Phase III Study of Ceralasertib Plus Durvalumab Versus Docetaxel in Patients With Non Small Cell Lung Cancer (NSCLC) Whose Disease Progressed On or After Prior Anti PD (L)1 Therapy And Platinum Based Chemotherapy	https://clinicaltrials.gov/study/NCT05450692
Lung	NCT05767892	YK-029A as First-Line Treatment Versus Platinum-Based Chemotherapy for Non-Small Cell Lung Cancer (NSCLC) With EGFR Exon 20 Insertion Mutations	https://clinicaltrials.gov/study/NCT05767892

© 2024 Hibbs SP et al. *JAMA Network Open*.

Lung	NCT05184712	Phase 3 Clinical Study of AK112 for NSCLC Patients	https://clinicaltrials.gov/study/NCT05184712
Lung	NCT05870319	A Phase III Study of SKB264 for EGFR Mutant NSCLC Patients	https://clinicaltrials.gov/study/NCT05870319
Lung	NCT05096663	Testing the Use of Combination Immunotherapy Treatment (N-803 [ALT-803] Plus Pembrolizumab) Against the Usual Treatment for Advanced Non-small Cell Lung Cancer (A Lung-MAP Treatment Trial)	https://clinicaltrials.gov/study/NCT05096663
Lung	NCT04996017	Atezolizumab Versus Placebo for the Adjuvant Treatment of Malignant Pleural Mesothelioma (Atezomeso)	https://clinicaltrials.gov/study/NCT04996017
Lung	NCT05378763	A Study of Poziotinib in Previously Treated Participants With Locally Advanced or Metastatic NSCLC Harboring HER2 Exon 20 Mutations	https://clinicaltrials.gov/study/NCT05378763
Lung	NCT05807893	Study to Evaluate the Safety and Efficacy of Serplulimab Plus Bevacizumab and Chemotherapy in NSCLC Patients With Brain Metastases	https://clinicaltrials.gov/study/NCT05807893
Lung	NCT05487391	A Study of QL1706 Combined With Platinum-containing Chemotherapy in Adjuvant Treatment of Stage II-IIIB Non-small Cell Lung Cancer After Complete Surgical Resection.	https://clinicaltrials.gov/study/NCT05487391
Lung	NCT05899608	Clinical Study of Ivonescimab for First-line Treatment of Metastatic Squamous NSCLC Patients	https://clinicaltrials.gov/study/NCT05899608
Lung	NCT05120349	A Global Study to Assess the Effects of Osimertinib in Participants With EGFRm Stage IA2-IA3 NSCLC Following Complete Tumour Resection	https://clinicaltrials.gov/study/NCT05120349
Lung	NCT05429463	Neoadjuvant Therapy of Sintilimab Combined With Chemotherapy for Resectable Squamous Cell NSCLCÔºàneoSCORE ,Ö°Ôºâ	https://clinicaltrials.gov/study/NCT05429463
Lung	NCT06008093	A Study to Investigate the Efficacy of Durvalumab Plus Tremelimumab in Combination With Chemotherapy Compared With Pembrolizumab in Combination With Chemotherapy in Metastatic Non-Small Cell Lung Cancer (NSCLC) Patients	https://clinicaltrials.gov/study/NCT06008093
Lung	NCT05170204	A Study Evaluating the Efficacy and Safety of Multiple Therapies in Cohorts of Participants With Locally Advanced, Unresectable, Stage III Non-Small Cell Lung Cancer (NSCLC)	https://clinicaltrials.gov/study/NCT05170204
Lung	NCT05015608	Study on Savolitinib Combined With Osimertinib in Treatment of Advanced NSCLC With MET Amplification	https://clinicaltrials.gov/study/NCT05015608
Lung	NCT05994339	Radiotherapy Combined With Almonertinib for Stage III EGFR-Mutated Lung Cancer	https://clinicaltrials.gov/study/NCT05994339
Lung	NCT05648071	First-Line Treatment for Advanced Non-squamous Non-Small-Cell Lung Cancer With Negative Driver Gene: a Single-center, Single-Arm Trial	https://clinicaltrials.gov/study/NCT05648071
Lung	NCT04597671	Durvalumab and Low-dose PCI vs Durvalumab and Observation in Radically Treated Patients With Stage III NSCLC (NVALT28)	https://clinicaltrials.gov/study/NCT04597671

© 2024 Hibbs SP et al. *JAMA Network Open*.

Lung	NCT05153239	Clinical Trial of Lurbinectedin as Single-agent or in Combination With Irinotecan Versus Topotecan or Irinotecan in Patients With Relapsed Small-cell Lung Cancer (LAGOON)	https://clinicaltrials.gov/study/NCT05153239
Lung	NCT05047250	A Study of Atezolizumab in High PD-L1 Expression, Chemotherapy-Na√Øve Patients With Stage IV Non-Squamous or Squamous Non-Small Cell Lung Cancer	https://clinicaltrials.gov/study/NCT05047250
Lung	NCT05840016	AK112 in Combination With Chemotherapy in Advanced Squamous Non-Small Cell Lung Cancer	https://clinicaltrials.gov/study/NCT05840016
Lung	NCT05499390	AK112 in Advanced Non-Small Cell Lung Cancer	https://clinicaltrials.gov/study/NCT05499390
Lung	NCT05673590	Utidelone Versus Docetaxel in Patients With Locally Advanced or Metastatic Non-Small Cell Lung Cancer	https://clinicaltrials.gov/study/NCT05673590
Lung	NCT05943795	A Clinical Study of SI-B001 Combined With Docetaxel in the Treatment of Non-small Cell Lung Adenocarcinoma and Lung Squamous Cell Carcinoma	https://clinicaltrials.gov/study/NCT05943795
Lung	NCT05211895	A Global Study to Assess the Effects of Durvalumab + Domvanalimab Following Concurrent Chemoradiation in Participants With Stage III Unresectable NSCLC	https://clinicaltrials.gov/study/NCT05211895
Lung	NCT05085028	A Randomised Open-label Phase III Trial of REduced Frequency Pembrolizumab immuNothErapy for First-line Treatment of Patients With Advanced Non-small Cell Lung Cancer (NSCLC)	https://clinicaltrials.gov/study/NCT05085028
Lung	NCT05061823	Bintrafusp Alfa Program Rollover Study	https://clinicaltrials.gov/study/NCT05061823
Lung	NCT05089734	Study of Sacituzumab Govitecan (SG) Versus Docetaxel in Participants With Advanced or Metastatic Non-Small Cell Lung Cancer (NSCLC)	https://clinicaltrials.gov/study/NCT05089734
Lung	NCT05298423	Study of Pembrolizumab/Vibostolimab (MK-7684A) in Combination With Concurrent Chemoradiotherapy Followed by Pembrolizumab/Vibostolimab Versus Concurrent Chemoradiotherapy Followed by Durvalumab in Participants With Stage III Non-small Cell Lung Cancer (MK-7684A-006/KEYVIBE-006)	https://clinicaltrials.gov/study/NCT05298423
Lung	NCT06031558	Phase III Study of SY-5007, a RET Inhibitor, in Patients With Locally Advanced or Metastatic RET Fusion-positive NSCLC	https://clinicaltrials.gov/study/NCT06031558
Lung	NCT05800223	Armatinib Alone or in Combination With SRT for Brain Metastases EGFR-mutated Non-small Cell Lung Cancer	https://clinicaltrials.gov/study/NCT05800223
Lung	NCT05224141	Pembrolizumab/Vibostolimab (MK-7684A) or Atezolizumab in Combination With Chemotherapy in First Line Treatment of Extensive-Stage Small Cell Lung Cancer (MK-7684A-008, KEYVIBE-008)	https://clinicaltrials.gov/study/NCT05224141
Lung	NCT05605613	PD-1 Antibody in Addition to BACE in Patients With NSCLC: A Randomised Controlled Trial	https://clinicaltrials.gov/study/NCT05605613
Lung	NCT05338970	HERTHENA-Lung02: A Study of Patritumab Deruxtecan Versus Platinum-based Chemotherapy in Metastatic or Locally Advanced EGFRm NSCLC After Failure of EGFR TKI Therapy	https://clinicaltrials.gov/study/NCT05338970

© 2024 Hibbs SP et al. *JAMA Network Open*.

Lung	NCT05132413	A Study of SHR-1701 Plus Bevacizumab and Chemotherapy in Non-Small-Cell-Lung-Cancer	https://clinicaltrials.gov/study/NCT05132413
Lung	NCT05624996	Testing the Addition of High Dose, Targeted Radiation to the Usual Treatment for Locally-Advanced Inoperable Non-small Cell Lung Cancer	https://clinicaltrials.gov/study/NCT05624996
Lung	NCT05106335	A Study to Evaluate Camrelizumab Combined With Famitinib as Subsequent Therapy in Patients With Advanced NSCLC	https://clinicaltrials.gov/study/NCT05106335
Lung	NCT05522660	Immunotherapy or Targeted Therapy With or Without Stereotactic Radiosurgery for Patients With Brain Metastases From Melanoma or Non-small Cell Lung Cancer	https://clinicaltrials.gov/study/NCT05522660
Lung	NCT05468489	To Evaluate Efficacy and Safety of Serplulimab + Chemotherapy (Carboplatin-Etoposide) in US Patients With ES-SCLC	https://clinicaltrials.gov/study/NCT05468489
Lung	NCT05555732	Datopotamab Deruxtecan (Dato-DXd) and Pembrolizumab With or Without Platinum Chemotherapy in 1L Non-Small Cell Lung Cancer (TROPION-Lung07)	https://clinicaltrials.gov/study/NCT05555732
Lung	NCT05973773	A Study of Zipalertinib and Chemotherapy Compared With Chemotherapy Alone in Patients With Advanced Non-Small Cell Lung Cancer With Epidermal Growth Factor Receptor (EGFR) Exon 20 Insertion.	https://clinicaltrials.gov/study/NCT05973773
Lung	NCT06043973	Almonertinib Combined With Anlotinib as First-line Treatment for Advanced Non-small Cell Lung Cance	https://clinicaltrials.gov/study/NCT06043973
Lung	NCT06062810	Pharmacogenomics IND EXEMPT SNP Clinical Study - Crizotinib and Single Nucleotide Polymorphisms	https://clinicaltrials.gov/study/NCT06062810
Lung	NCT04928846	A Study to Assess Disease Activity and Adverse Events of Intravenous (IV) Telisotuzumab Vedotin Compared to IV Docetaxel in Adult Participants With Previously Treated Non-Squamous Non-Small Cell Lung Cancer (NSCLC)	https://clinicaltrials.gov/study/NCT04928846
Lung	NCT04819100	A Study of Selpercatinib After Surgery or Radiation in Participants With Non-Small Cell Lung Cancer (NSCLC)	https://clinicaltrials.gov/study/NCT04819100
Lung	NCT05223647	Chemo-immunotherapy Plus Thoracic Radiotherapy in Extensive Stage Small-cell Lung Cancer	https://clinicaltrials.gov/study/NCT05223647
Lung	NCT06095583	A Study to Assess Toripalimab Alone or in Combination With Tifcemalimab as Consolidation Therapy in Patients With Limited-stage Small Cell Lung Cancer (LS-SCLC)	https://clinicaltrials.gov/study/NCT06095583
Lung	NCT05341583	Ensartinib as Adjuvant Treatment in Anaplastic Lymphoma Kinase (ALK) Positive Non-small Cell Lung Cancer	https://clinicaltrials.gov/study/NCT05341583
Lung	NCT05718167	TQB2450 Injection Combined With Chemotherapy Followed by Sequential Combination With Anlotinib Hydrochloride Capsule for First-line Treatment of Advanced Squamous Non-small Cell Lung Cancer.	https://clinicaltrials.gov/study/NCT05718167
Lung	NCT05623267	Sugemalimab as Consolidation Therapy in Patients With LS-SCLC Following cCRT or sCRT	https://clinicaltrials.gov/study/NCT05623267

© 2024 Hibbs SP et al. *JAMA Network Open*.

Lung	NCT05918302	Efficacy and Safety of Radiotherapy Compared to Everolimus in Somatostatin Receptor Positive Neuroendocrine Tumors of the Lung and Thymus.	https://clinicaltrials.gov/study/NCT05918302
Lung	NCT05255302	De-escalation Immunotherapy mAintenance Duration Trial for Stage IV Lung Cancer Patients With Disease Control After Chemo-immunotherapy Induction	https://clinicaltrials.gov/study/NCT05255302
Lung	NCT05633602	Ramucirumab Plus Pembrolizumab vs Usual Care for Treatment of Stage IV or Recurrent Non-Small Cell Lung Cancer Following Immunotherapy, Pragmatica-Lung Study	https://clinicaltrials.gov/study/NCT05633602
Lung	NCT05768178	DETERMINE Trial Treatment Arm 05: Vemurafenib in Combination With Cobimetinib in Adult Patients With BRAF Positive Cancers.	https://clinicaltrials.gov/study/NCT05768178
Lung	NCT05215340	Study of Dato-DXd Plus Pembrolizumab vs Pembrolizumab Alone in the First-line Treatment of Subjects With Advanced or Metastatic NSCLC Without Actionable Genomic Alterations	https://clinicaltrials.gov/study/NCT05215340
Lung	NCT06080776	SH-1028 Tablets Versus Placebo as Adjuvant Therapy in Resected Stage II-IIIB NSCLC With Sensitizing EGFR Mutations	https://clinicaltrials.gov/study/NCT06080776
Lung	NCT04786964	Study of Pemetrexed+Platinum Chemotherapy With or Without Cosibelimab (CK-301) in First Line Metastatic Non-squamous Non-Small Cell Lung Cancer	https://clinicaltrials.gov/study/NCT04786964
Lung	NCT05493501	Aumolertinib With Chemotherapy or Alone Compared With Osimertinib in Patients With Epidermal Growth Factor Receptor-Mutant Non-Small Cell Lung Cancer	https://clinicaltrials.gov/study/NCT05493501
Lung	NCT05353257	A Study to Evaluate the Efficacy and Safety of Serplulimab in Combination With Chemotherapy and Concurrent Radiotherapy in Patients With Limited-Stage Small Cell Lung Cancer	https://clinicaltrials.gov/study/NCT05353257
Lung	NCT05221840	A Global Study to Assess the Effects of Durvalumab With Oleclumab or Durvalumab With Monalizumab Following Concurrent Chemoradiation in Patients With Stage III Unresectable Non-Small Cell Lung Cancer	https://clinicaltrials.gov/study/NCT05221840
Lung	NCT05661240	Phase II/III Clinical Study of Tumor Fields (EFE-P100) Combined with Docetaxel in the Treatment of stage IV Non-small Cell Lung Cancer Patients with Disease Progression After Platinum-based Chemotherapy and Anti-programmed Death 1 (PD-1)/Programmed Cell Death-Ligand 1(PD-L1) Antibody Treatment	https://clinicaltrials.gov/study/NCT05661240
Lung	NCT05261399	Savolitinib Plus Osimertinib Versus Platinum-based Doublet Chemotherapy in Participants With Non-Small Cell Lung Cancer Who Have Progressed on Osimertinib Treatment	https://clinicaltrials.gov/study/NCT05261399
Lung	NCT04988295	A Study of Amivantamab and Lazertinib in Combination With Platinum-Based Chemotherapy Compared With Platinum-Based Chemotherapy in Patients With Epidermal Growth Factor Receptor (EGFR)-Mutated Locally Advanced or Metastatic Non- Small Cell Lung Cancer After Osimertinib Failure	https://clinicaltrials.gov/study/NCT04988295

© 2024 Hibbs SP et al. *JAMA Network Open*.

Lung	NCT05692999	Maintenance Pembrolizumab at Usual or Low doSE in Non-squamous Lung Cancer: a Non-inferiority Study	https://clinicaltrials.gov/study/NCT05692999
Lung	NCT04774380	Study of Durvalumab in Combination With Platinum and Etoposide for the First Line Treatment of Patients With Extensive-stage Small Cell Lung Cancer	https://clinicaltrials.gov/study/NCT04774380
Lung	NCT05566041	A Phase 3, Controlled, Open-label, Global Randomized Study of RRx-001 With a Platinum Doublet or a Platinum Doublet in Small Cell Lung Cancer	https://clinicaltrials.gov/study/NCT05566041
Lung	NCT04929041	Testing the Addition of Radiation Therapy to the Usual Treatment (Immunotherapy With or Without Chemotherapy) for Stage IV Non-Small Cell Lung Cancer Patients Who Are PD-L1 Negative	https://clinicaltrials.gov/study/NCT04929041
Lung	NCT06041776	Adjuvant Befotertinib in Stage IB-IIIB Non-small Cell Lung Cancer With Positive EGFR Sensitive Mutations	https://clinicaltrials.gov/study/NCT06041776
Lung	NCT05226598	Study of Pembrolizumab/Vibostolimab Coformulation (MK-7684A) in Combination With Chemotherapy Versus Pembrolizumab Plus Chemotherapy in Participants With Metastatic Non-Small Cell Lung Cancer (MK-7684A-007/KEYVIBE-007)	https://clinicaltrials.gov/study/NCT05226598
Lung	NCT05382728	Phase III Study of TY-9591 in Patients With Locally Advanced or Metastatic Non-small Cell Lung Cancer (FLETEO)	https://clinicaltrials.gov/study/NCT05382728
Lung	NCT05204628	A Study to Evaluate and Compare the Efficacy and Safety of XZP-3621 Versus Crizotinib	https://clinicaltrials.gov/study/NCT05204628
Lung	NCT05020769	SI-B001 Combined With Osimertinib Mesylate Tablets in the Treatment of Recurrent Metastatic Non-small Cell Lung Cancer.	https://clinicaltrials.gov/study/NCT05020769
Lung	NCT05388669	A Study of Lazertinib With Subcutaneous Amivantamab Compared With Intravenous Amivantamab in Participants With Epidermal Growth Factor Receptor (EGFR)-Mutated Advanced or Metastatic Non-small Cell Lung Cancer	https://clinicaltrials.gov/study/NCT05388669
Colorectal	NCT05236972	PACE: PD-1 Antibody For dMMR/MSI-H Stage III Colorectal Cancer	https://clinicaltrials.gov/study/NCT05236972
Colorectal	NCT05534087	Platform Study of Circulating Tumor DNA Directed Adjuvant Chemotherapy in Colon Cancer (KCSG CO22-12)	https://clinicaltrials.gov/study/NCT05534087
Colorectal	NCT05815082	ctDNA-guided Adjuvant Chemotherapy in Liver Metastasis of Colorectal Cancer	https://clinicaltrials.gov/study/NCT05815082
Colorectal	NCT05064059	A Study of Coformulated Favezelimab/Pembrolizumab (MK-4280A) Versus Standard of Care in Subjects With Previously Treated Metastatic PD-L1 Positive Colorectal Cancer (MK-4280A-007)	https://clinicaltrials.gov/study/NCT05064059
Colorectal	NCT05482516	Evaluating Novel Therapies in ctDNA Positive GI Cancers	https://clinicaltrials.gov/study/NCT05482516
Colorectal	NCT05378867	A Study Assessing the Interchangeability Between TRS003 and Bevacizumab¬Æ For CRC	https://clinicaltrials.gov/study/NCT05378867
Colorectal	NCT05797467	Adjuvant Chemotherapy Combined With Targeted Therapy or Not in the T3-4N2 Colorectal Cancer Patients	https://clinicaltrials.gov/study/NCT05797467

© 2024 Hibbs SP et al. *JAMA Network Open*.

Colorectal	NCT05768503	Comparing Chidamide+Sintilimab+Bev With Standard Second-line FOLFIRI+Bev in Advanced MSS/pMMR mCRC	https://clinicaltrials.gov/study/NCT05768503
Colorectal	NCT05673512	To Evaluate IAH0968 in Combination With CAPEOX in HER2-positive Metastatic Colorectal Cancer	https://clinicaltrials.gov/study/NCT05673512
Colorectal	NCT05253651	A Study of Tucatinib With Trastuzumab and mFOLFOX6 Versus Standard of Care Treatment in First-line HER2+ Metastatic Colorectal Cancer	https://clinicaltrials.gov/study/NCT05253651
Colorectal	NCT05409417	Exploratory Study on Combined Conversion Immunotherapy for Liver Metastasis of MSS Type Initial Unresectable Colorectal Cancer Based on Gene Status	https://clinicaltrials.gov/study/NCT05409417
Colorectal	NCT05861505	COLLISION RELAPSE Trial	https://clinicaltrials.gov/study/NCT05861505
Colorectal	NCT05328908	A Study of Nivolumab-relatlimab Fixed-dose Combination Versus Regorafenib or TAS-102 in Participants With Later-lines of Metastatic Colorectal Cancer	https://clinicaltrials.gov/study/NCT05328908
Colorectal	NCT05600309	A Study of Coformulated Favezelimab/Pembrolizumab (MK-4280A) Versus Standard of Care in Subjects With Previously Treated Metastatic PD-L1 Positive Colorectal Cancer (MK-4280A-007)-China Extension Study	https://clinicaltrials.gov/study/NCT05600309
Colorectal	NCT05374252	Chemoradiotherapy Combined With or Without PD-1 Blockade in Anal Canal Squamous Carcinoma Patients	https://clinicaltrials.gov/study/NCT05374252
Colorectal	NCT04749108	Study Evaluating the Tailored Management of Locally-advanced Rectal Carcinoma	https://clinicaltrials.gov/study/NCT04749108
Colorectal	NCT05008809	Post-resection/Ablation Chemotherapy in Patients With Metastatic Colorectal Cancer (FIRE-9 - PORT / AIO-KRK-0418)	https://clinicaltrials.gov/study/NCT05008809
Colorectal	NCT05794971	Regorafenib Combined With Irinotecan Drug-Eluting Beads for Colorectal Cancer Liver Metastases	https://clinicaltrials.gov/study/NCT05794971
Colorectal	NCT05215379	Neoadjuvant Chemoradiation Therapy Combined With Immunotherapy for MSS Ultra-low Rectal Cancer	https://clinicaltrials.gov/study/NCT05215379
Colorectal	NCT02942706	Cetuximab Maintenance Treatment Versus Continuation After Induction Therapy in mCRC	https://clinicaltrials.gov/study/NCT02942706
Colorectal	NCT05710406	Testing the Use of BRAF-Targeted Therapy After Surgery and Usual Chemotherapy for BRAF-Mutated Colon Cancer	https://clinicaltrials.gov/study/NCT05710406
Colorectal	NCT05948072	Cetuximab+mFOLFOX6 VS. mFOLFOX6 Alone in RAS/BRAF Wild Type Patients With High-Risk Resectable CRLM	https://clinicaltrials.gov/study/NCT05948072
Colorectal	NCT05239741	Study of Pembrolizumab (MK-3475) Versus Chemotherapy in Chinese Participants With Stage IV Colorectal Cancer (MK-3475-C66)	https://clinicaltrials.gov/study/NCT05239741
Colorectal	NCT05770102	DETERMINE Trial Treatment Arm 02: Atezolizumab in Adult, Teenage/Young Adults and Paediatric Patients With Cancers With High Tumour Mutational Burden (TMB) or Microsatellite Instability-high (MSI-high) or Proven Constitutional Mismatch Repair Deficiency (CMMRD) Disposition	https://clinicaltrials.gov/study/NCT05770102

© 2024 Hibbs SP et al. *JAMA Network Open*.

Colorectal	NCT03678428	FUDR/Oxaliplatin HAI Plus Irinotecan vs. FOLFOXIRI Chemotherapy in Treating Initially Unresectable CRCLM	https://clinicaltrials.gov/study/NCT03678428
Colorectal	NCT05171660	Combination With Sintilimab and XELOX+Bevacizumab as 1st Line Therapy in RAS-mutant Metastatic Colorectal Cancer	https://clinicaltrials.gov/study/NCT05171660
Colorectal	NCT05652894	A Study of HX008 Compared to Chemotherapy in the First-Line Treatment of Subjects With MSI-H/dMMR Metastatic Colorectal Cancer	https://clinicaltrials.gov/study/NCT05652894
Colorectal	NCT05223673	Phase 3 Study of Futuximab/Modotuximab in Combination With Trifluridine/Tipiracil Versus Trifluridine/Tipiracil Single Agent in Participants With Previously Treated Metastatic Colorectal Cancer	https://clinicaltrials.gov/study/NCT05223673
Colorectal	NCT05699746	CAPEOX vs Observation in Colorectal Cancer Patients With Positive MRD	https://clinicaltrials.gov/study/NCT05699746
Colorectal	NCT06017583	Neoadjuvant Chemotherapy With PD-1 Inhibitors Combined With SIB-IMRT in the Treatment of Locally Advanced Rectal Cancer	https://clinicaltrials.gov/study/NCT06017583
Colorectal	NCT05855200	Study of Perioperative Dostarlimab in Participants With Untreated T4N0 or Stage III dMMR/MSI-H Resectable Colon Cancer	https://clinicaltrials.gov/study/NCT05855200
Colorectal	NCT05194878	Neoadjuvant FOLFOXIRI Versus Immediate Surgery for Stage II and III Colon Cancers	https://clinicaltrials.gov/study/NCT05194878
Colorectal	NCT05954078	Circulating Tumor DNA Methylation Guided Postoperative Adjuvant Chemotherapy for High-risk Stage II/III Colorectal Cancer	https://clinicaltrials.gov/study/NCT05954078
Colorectal	NCT05141721	A Study of a Patient-Specific Neoantigen Vaccine in Combination With Immune Checkpoint Blockade for Patients With Metastatic Colorectal Cancer	https://clinicaltrials.gov/study/NCT05141721
Colorectal	NCT05198934	Sotorasib and Panitumumab Versus Investigator's Choice for Participants With Kirsten Rat Sarcoma (KRAS) p.G12C Mutation	https://clinicaltrials.gov/study/NCT05198934
Colorectal	NCT05484024	Short-course Radiotherapy Followed by Chemotherapy and PD-1 Inhibitor for Locally Advanced Rectal Cancer	https://clinicaltrials.gov/study/NCT05484024
Colorectal	NCT05425940	Study of XL092 + Atezolizumab vs Regorafenib in Subjects With Metastatic Colorectal Cancer	https://clinicaltrials.gov/study/NCT05425940
Colorectal	NCT05646511	Total Neoadjuvant Therapy of SCRT+CAPOX vs SCRT+CAPOXIRI for Locally Advanced Rectal Cancer (ENSEMBLE)	https://clinicaltrials.gov/study/NCT05646511
Colorectal	NCT05945901	A Phase II/III Study of HR070803 in Combination With Oxaliplatin, 5-fluorouracil, Calcium Folinate and Bevacizumab Versus FOLFOX in Combination With Bevacizumab for First-line Treatment of Advanced Colorectal Cancer	https://clinicaltrials.gov/study/NCT05945901
Colorectal	NCT05462613	Regorafenib With Low-dose Chemotherapies and Aspirin Followed by Standard Chemotherapies in Metastatic Colorectal Cancer	https://clinicaltrials.gov/study/NCT05462613
Colorectal	NCT05174169	Colon Adjuvant Chemotherapy Based on Evaluation of Residual Disease	https://clinicaltrials.gov/study/NCT05174169
Colorectal	NCT05427669	Adjuvant mFOLFOXIRI vs. mFOLFOX6 in MRD Positive Stage II-III Colorectal Cancer (AFFORD)	https://clinicaltrials.gov/study/NCT05427669

© 2024 Hibbs SP et al. *JAMA Network Open*.

Melanoma	NCT04901988	Circulating Tumour DNA guidEd Therapy for Stage IIB/C mElanoma After surgiCal resecTION	https://clinicaltrials.gov/study/NCT04901988
Melanoma	NCT05647954	A Study of HX008 Plus Transcatheter Arterial Chemoembolization (TACE) in the First-Line Treatment of Subjects With Stage IV (M1c) Melanoma That is Metastatic to the Liver	https://clinicaltrials.gov/study/NCT05647954
Melanoma	NCT05155254	IO102-IO103 in Combination With Pembrolizumab Versus Pembrolizumab Alone in Advanced Melanoma (IOB-013 / KN-D18)	https://clinicaltrials.gov/study/NCT05155254
Melanoma	NCT05907122	A Study to Evaluate Similarity of ABP 206 Compared With OPDIVO¬Æ (Nivolumab) in Subjects With Resected Melanoma	https://clinicaltrials.gov/study/NCT05907122
Melanoma	NCT05868707	OH2 Injection in Melanoma	https://clinicaltrials.gov/study/NCT05868707
Melanoma	NCT06054555	A Study to Evaluate ABP 206 Compared With OPDIVO¬Æ (Nivolumab) in Subjects With Unresectable or Metastatic Melanoma	https://clinicaltrials.gov/study/NCT06054555
Melanoma	NCT05732805	A Clinical Study of BCD-217 (Nurulimab + Prolgolimab) Followed by Anti-PD-1 Compared to Anti-PD-1 Monotherapy as First-Line Treatment in Subjects With Unresectable/Metastatic Melanoma	https://clinicaltrials.gov/study/NCT05732805
Melanoma	NCT05608291	A Trial to See if the Combination of Fianlimab With Cemiplimab Works Better Than Pembrolizumab for Preventing or Delaying Melanoma From Coming Back After it Has Been Removed With Surgery	https://clinicaltrials.gov/study/NCT05608291
Melanoma	NCT05727904	Study to Investigate Lifileucel Regimen Plus Pembrolizumab Compared With Pembrolizumab Alone in Participants With Untreated Advanced Melanoma.	https://clinicaltrials.gov/study/NCT05727904
Melanoma	NCT06008106	Comparing Tunlametinib Capsules and Combination Chemotherapy in Advanced NRAS-mutant Melanoma	https://clinicaltrials.gov/study/NCT06008106
Melanoma	NCT03928275	The Response to Intralesional IL-2 and/or BCG Treatment for Cutaneous Metastatic Melanoma	https://clinicaltrials.gov/study/NCT03928275
Melanoma	NCT05783882	Prolgolimab 250 mg Q3W in Patients With Unresectable or Metastatic Melanoma	https://clinicaltrials.gov/study/NCT05783882
Melanoma	NCT05665595	A Study of Adjuvant Pembrolizumab/Vibostolimab (MK-7684A) Versus Pembrolizumab for Resected High-Risk Melanoma in Participants With High-Risk Stage II-IV Melanoma (MK-7684A-010/KEYVIBE-010)	https://clinicaltrials.gov/study/NCT05665595
Melanoma	NCT05352672	Clinical Study of Fianlimab in Combination With Cemiplimab in Adolescent and Adult Patients With Previously Untreated Unresectable Locally Advanced or Metastatic Melanoma	https://clinicaltrials.gov/study/NCT05352672
Melanoma	NCT05987332	IDE196 (Darovasertib) in Combination With Crizotinib as First-line Therapy in Metastatic Uveal Melanoma	https://clinicaltrials.gov/study/NCT05987332
Melanoma	NCT05751928	A Study of Neoadjuvant Therapy With BCD-217 (Nurulimab + Prolgolimab) in Patients With Resectable Stage III Skin Melanoma	https://clinicaltrials.gov/study/NCT05751928

© 2024 Hibbs SP et al. *JAMA Network Open*.

Melanoma	NCT05986331	Clinical Study of the Efficacy and Safety of BCD-201 and Keytruda in Subjects With Advanced Melanoma	https://clinicaltrials.gov/study/NCT05986331
Melanoma	NCT05297565	A Study to Compare Nivolumab Administered Subcutaneously vs Intravenous in Melanoma Participants Following Complete Resection	https://clinicaltrials.gov/study/NCT05297565
Melanoma	NCT05549297	Tebentafusp Regimen Versus Investigator's Choice in Previously Treated Advanced Melanoma (TEBE-AM)	https://clinicaltrials.gov/study/NCT05549297
Melanoma	NCT05789043	Camrelizumab in Combination With Apatinib and Temozolomide as First-line Treatment in Advanced Acral Melanoma	https://clinicaltrials.gov/study/NCT05789043
Melanoma	NCT05022901	An Open-Label Expanded Access Study of the Melphalan/Hepatic Delivery System (HDS) in Patients With Hepatic Dominant Ocular Melanoma	https://clinicaltrials.gov/study/NCT05022901
Melanoma	NCT05933577	A Clinical Study of V940 Plus Pembrolizumab in People With High-Risk Melanoma (V940-001)	https://clinicaltrials.gov/study/NCT05933577
Melanoma	NCT05270044	Adjuvant Encorafenib and Binimetinib in High-risk Stage II Melanoma With a BRAF Mutation.	https://clinicaltrials.gov/study/NCT05270044
Melanoma	NCT05625399	A Study of Subcutaneous Nivolumab + Relatlimab Fixed-dose Combination (FDC) in Previously Untreated Metastatic or Unresectable Melanoma	https://clinicaltrials.gov/study/NCT05625399

© 2024 Hibbs SP et al. *JAMA Network Open*.

eTable 4. SACT regimen cohort. Listings are shown verbatim as listed in NCCN Guidelines except for minor clarifying corrections.

Cancer Type	NCCN regimen	Category of SACT
Prostate	EBRT/LHRH agonist	Hormonal therapy
Prostate	EBRT/LHRH agonist/first-generation antiandrogen	Hormonal therapy
Prostate	EBRT/Degarelix	Hormonal therapy
Prostate	EBRT/Relugolix	Hormonal therapy
Prostate	EBRT/LHRH agonist/abiraterone or fine-particle abiraterone	Hormonal therapy
Prostate	EBRT/Degarelix/abiraterone or fine-particle abiraterone	Hormonal therapy
Prostate	LHRH Agonist	Hormonal therapy
Prostate	LHRH antagonist (degarelix)	Hormonal therapy
Prostate	LHRH antagonist (relugolix)	Hormonal therapy
Prostate	LHRH agonist/first-generation antiandrogen	Hormonal therapy
Prostate	LHRH agonist/abiraterone or fine-particle abiraterone	Hormonal therapy
Prostate	LHRH antagonist (degarelix)/abiraterone or fine-particle abiraterone	Hormonal therapy
Melanoma	Pembrolizumab	Targeted Therapy
Melanoma	Nivolumab	Targeted Therapy
Melanoma	Dabrafenib/trametinib	Targeted Therapy
Breast	Dose-dense AC (doxorubicin/cyclophosphamide) followed or preceded by paclitaxel every 2 weeks	Chemotherapy
Breast	Dose-dense AC (doxorubicin/cyclophosphamide) followed or preceded by weekly paclitaxel	Chemotherapy
Breast	TC (docetaxel and cyclophosphamide)	Chemotherapy
Breast	Olaparib, if germline BRCA1/2 mutations	Targeted Therapy
Breast	Preoperative pembrolizumab/carboplatin/paclitaxel, followed by preoperative pembrolizumab/cyclophosphamide/doxorubicin or epirubicin	Chemotherapy; Targeted Therapy
Breast	Capecitabine	Chemotherapy
Breast	Paclitaxel/trastuzumab	Chemotherapy; Targeted Therapy
Breast	TCH (docetaxel/carboplatin/trastuzumab)	Chemotherapy; Targeted Therapy
Breast	TCHP (docetaxel/carboplatin/trastuzumab)/pertuzumab	Chemotherapy; Targeted Therapy

© 2024 Hibbs SP et al. *JAMA Network Open*.

Breast	Ado-trastuzumab emtansine	Targeted Therapy
Breast	Tamoxifen	Hormonal Therapy
Breast	Anastrazole	Hormonal Therapy
Breast	Letrozole	Hormonal Therapy
Breast	Exemestane	Hormonal Therapy
Colorectal	FOLFOX (3 months)	Chemotherapy
Colorectal	FOLFOX (6 months)	Chemotherapy
Colorectal	CapeOx (3 months)	Chemotherapy
Colorectal	CapeOx (6 months)	Chemotherapy
Colorectal	Capecitabine (6 months)	Chemotherapy
Colorectal	5-FU/leucovorin (6months)	Chemotherapy
Colorectal	Folinic Acid, Flurouracil, irinotecan and Oxaliplatin (FOLFIRINOX)	Chemotherapy
Colorectal	Nivolumab	Targeted Therapy
Colorectal	Nivolumab/ipilimumab	Targeted Therapy
Colorectal	Pembrolizumab	Targeted Therapy
Colorectal	Dostarlimab-gxly	Targeted Therapy
Lung	Cisplatin 50 mg/m2 days 1 and 8; vinorelbine 25 mg/m2 days 1, 8, 15, 22, every 28 days for 4 cycles	Chemotherapy
Lung	Cisplatin 100 mg/m2 day 1; vinorelbine 30 mg/m2 days 1, 8, 15, 22, every 28 days for 4 cycles	Chemotherapy
Lung	Cisplatin 75–80 mg/m2 day 1; vinorelbine 25–30 mg/m2 days 1 + 8, every 21 days for 4 cycles	Chemotherapy
Lung	Cisplatin 100 mg/m2 day 1; etoposide 100 mg/m2 days 1–3, every 28 days for 4 cycles	Chemotherapy
Lung	Cisplatin 75 mg/m2 day 1; gemcitabine 1250 mg/m2 days 1, 8, every 21 days for 4 cycles	Chemotherapy
Lung	Cisplatin 75 mg/m2 day 1; docetaxel 75 mg/m2 day 1 every 21 days for 4 cycles	Chemotherapy
Lung	Cisplatin 75 mg/m2 day 1, pemetrexed 500 mg/m2 day 1 for nonsquamous every 21 days for 4 cycles	Chemotherapy
Lung	Carboplatin AUC 6 day 1, paclitaxel 200 mg/m2 day 1, every 21 days for 4 cycles	Chemotherapy
Lung	Carboplatin AUC 5 day 1, gemcitabine 1000 mg/m2 days 1, 8, every 21 days for 4 cycles	Chemotherapy
Lung	Carboplatin AUC 5 day 1, pemetrexed 500 mg/m2 day 1 for nonsquamous every 21 days for 4 cycles	Chemotherapy

© 2024 Hibbs SP et al. *JAMA Network Open*.

Lung	Nivolumab/gemcitabine (squamous)/(carboplatin or cisplatin)	Chemotherapy; Targeted Therapy
Lung	Nivolumab/paclitaxel/(carboplatin or cisplatin)	Chemotherapy; Targeted Therapy
Lung	Nivolumab/pemetrexed (nonsquamous)/(carboplatin or cisplatin)	Chemotherapy; Targeted Therapy
Lung	Pembrolizumab/cisplatin/gemcitabine (squamous)	Chemotherapy; Targeted Therapy
Lung	Pembrolizumab/cisplatin/pemetrexed (nonsquamous)	Chemotherapy; Targeted Therapy
Lung	Osimertinib	Targeted Therapy
Lung	Pembrolizumab	Targeted Therapy
Lung	Cisplatin/etoposide with concurrent thoracic RT	Chemotherapy
Lung	Carboplatin AUC 5/pemetrexed with concurrent thoracic RT (nonsquamous)	Chemotherapy
Lung	Cisplatin/pemetrexed with concurrent thoracic RT (nonsquamous)	Chemotherapy
Lung	Cisplatin/pemetrexed with concurrent thoracic RT (nonsquamous) + additional 4 cycles of pemetrexed	Chemotherapy
Lung	Paclitaxel/carboplatin AUC 2 with concurrent thoracic RT	Chemotherapy
Lung	Paclitaxel/carboplatin AUC 2 with concurrent thoracic RT + additional 2 cycles of paclitaxel and carboplatin AUC 6	Chemotherapy
Lung	Cisplatin (75 mg)/etoposide (100 mg) + RT	Chemotherapy
Lung	Cisplatin (60 mg)/etoposide (120 mg) + RT	Chemotherapy
Lung	Carboplatin/etoposide + RT	Chemotherapy
Lung	Cisplatin (75 mg)/etoposide (100 mg)	Chemotherapy
Lung	Cisplatin 25 mg/m2 and etoposide 100 mg/m2	Chemotherapy
Lung	Cisplatin (60 mg)/etoposide (120 mg)	Chemotherapy
Lung	Carboplatin/etoposide	Chemotherapy
Lung	Cisplatin (80 mg)/etoposide (100 mg) + RT	Chemotherapy

© 2024 Hibbs SP et al. *JAMA Network Open*.

eFigure 1. CCT cohort search results and exclusions. Trials that included more than one cancer type were included in each applicable cancer type.

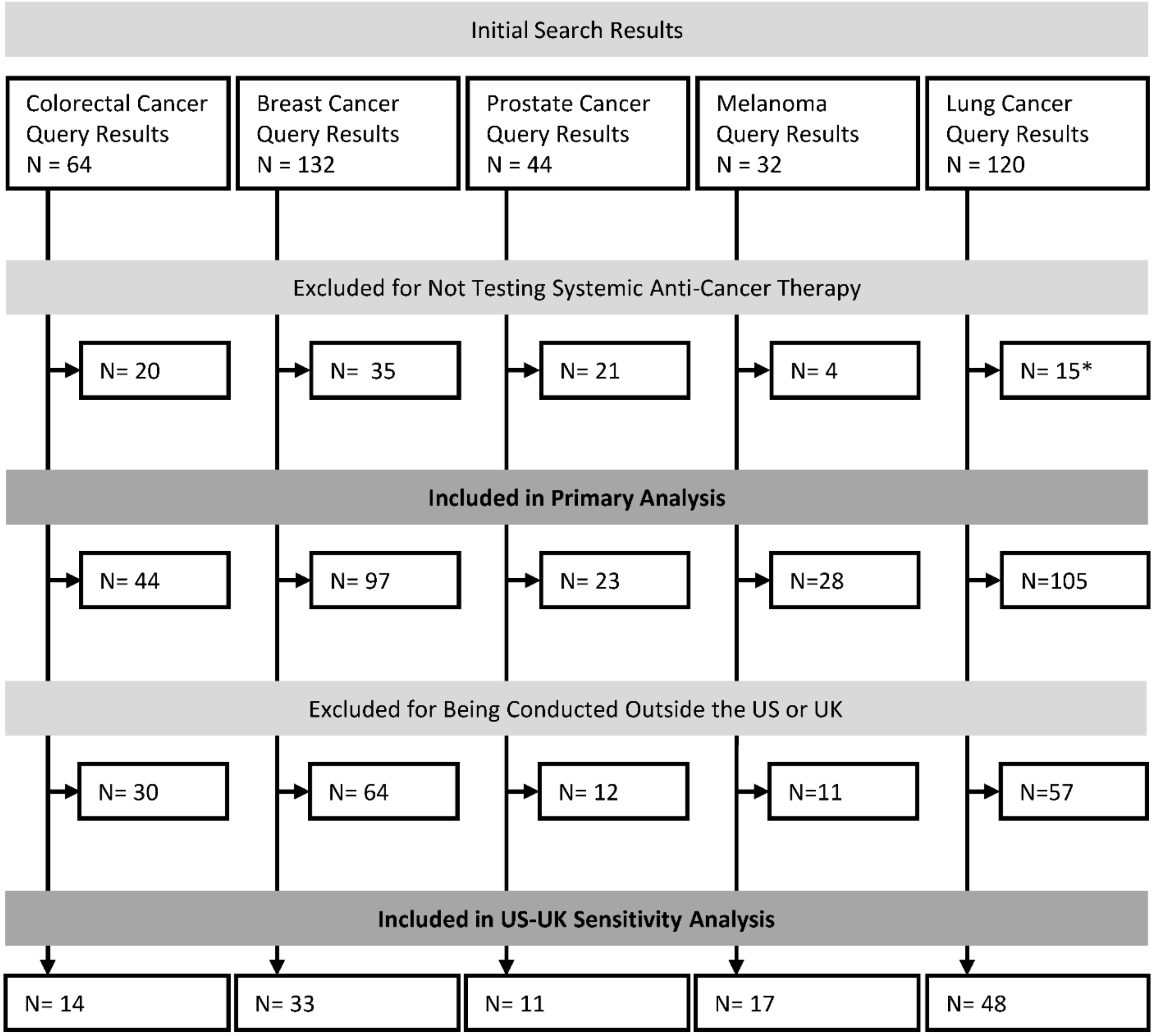

*One trial excluded for lack of available eligibility criteria

© 2024 Hibbs SP et al. *JAMA Network Open*.

eFigure 2. SACT cohort search results and exclusions.

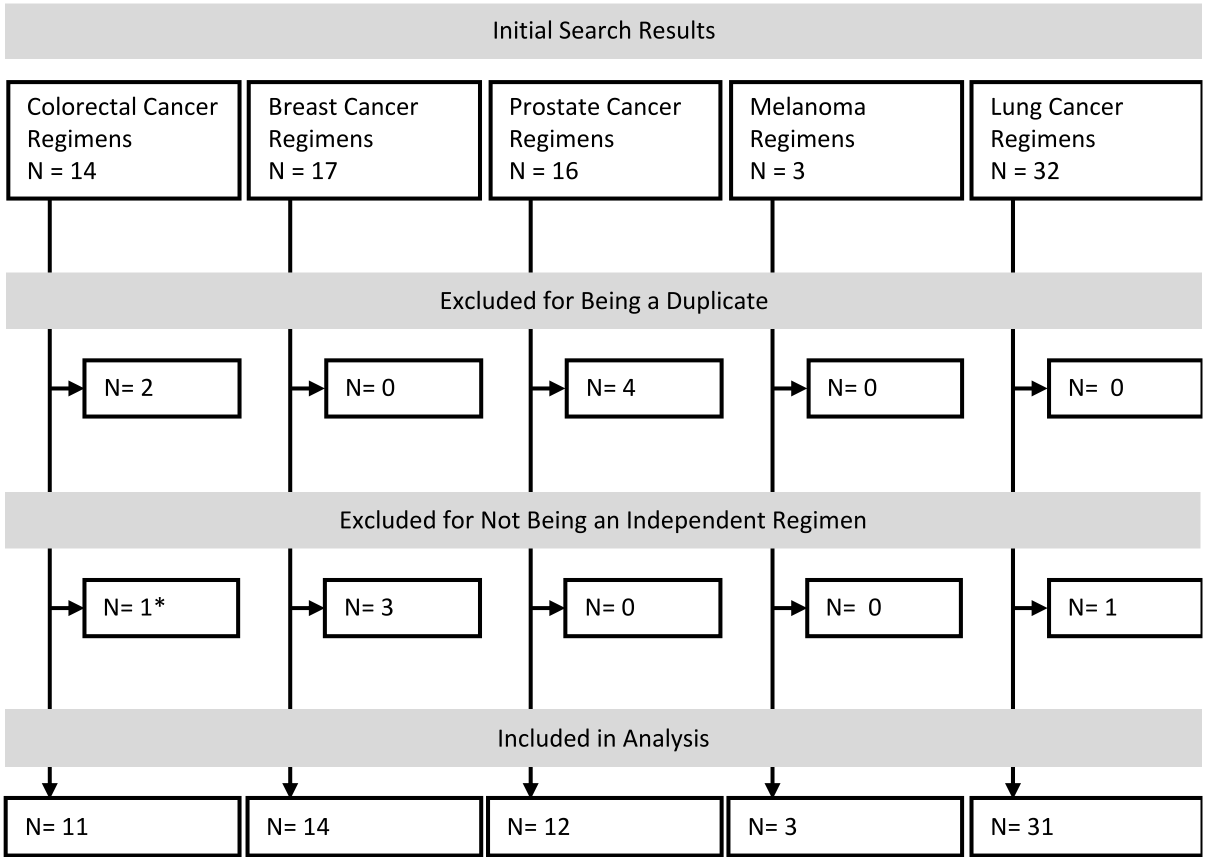

*Excluded for discrepancies in NCCN Preferred regimen listing

© 2024 Hibbs SP et al. *JAMA Network Open*.

eFigure 3. Proportions of clinical trials that exclude patients for ANC values within the DANC reference range by type of therapy and type of restriction. Trials listed within chemotherapy are all those including cytotoxic chemotherapy (N=142), those listed as targeted therapy did not also test chemotherapy (N=123), those listed as hormonal therapy did not also test chemotherapy or targeted therapy (N=24).

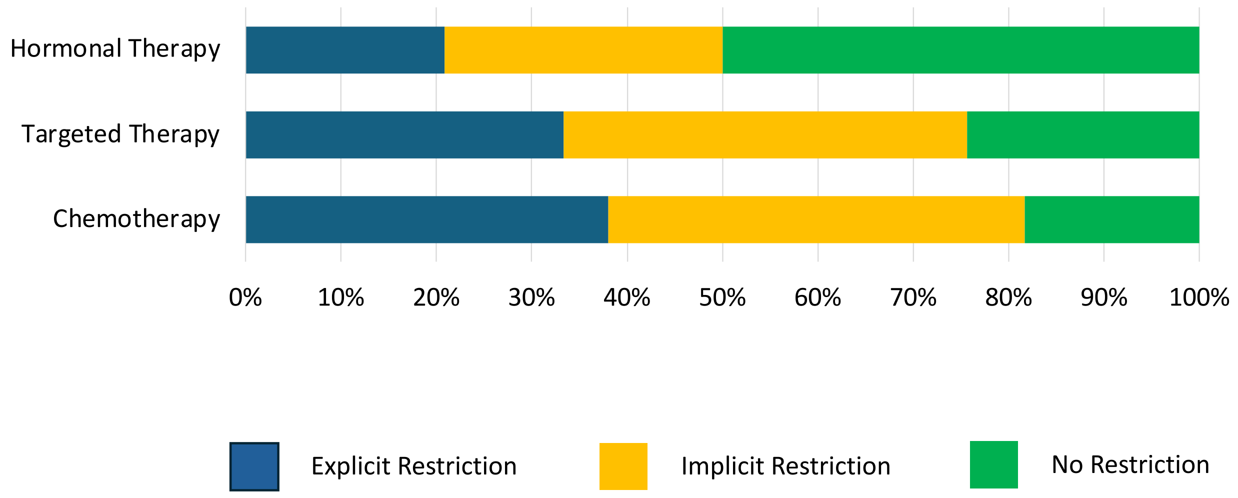

© 2024 Hibbs SP et al. *JAMA Network Open*.

eFigure 4. Proportions of clinical trials within the US and UK that exclude patients for ANC values within the DANC reference range by cancer type and type of restriction. Bars representing individual cancer types show all trials that include that cancer type (11 prostate, 17 melanoma, 33 breast, 14 colorectal, 48 lung), Overall Crude values are for unique trials (N=116), and Overall Weighted values weight each cancer type equally and include duplicates across cancer types (N=123).

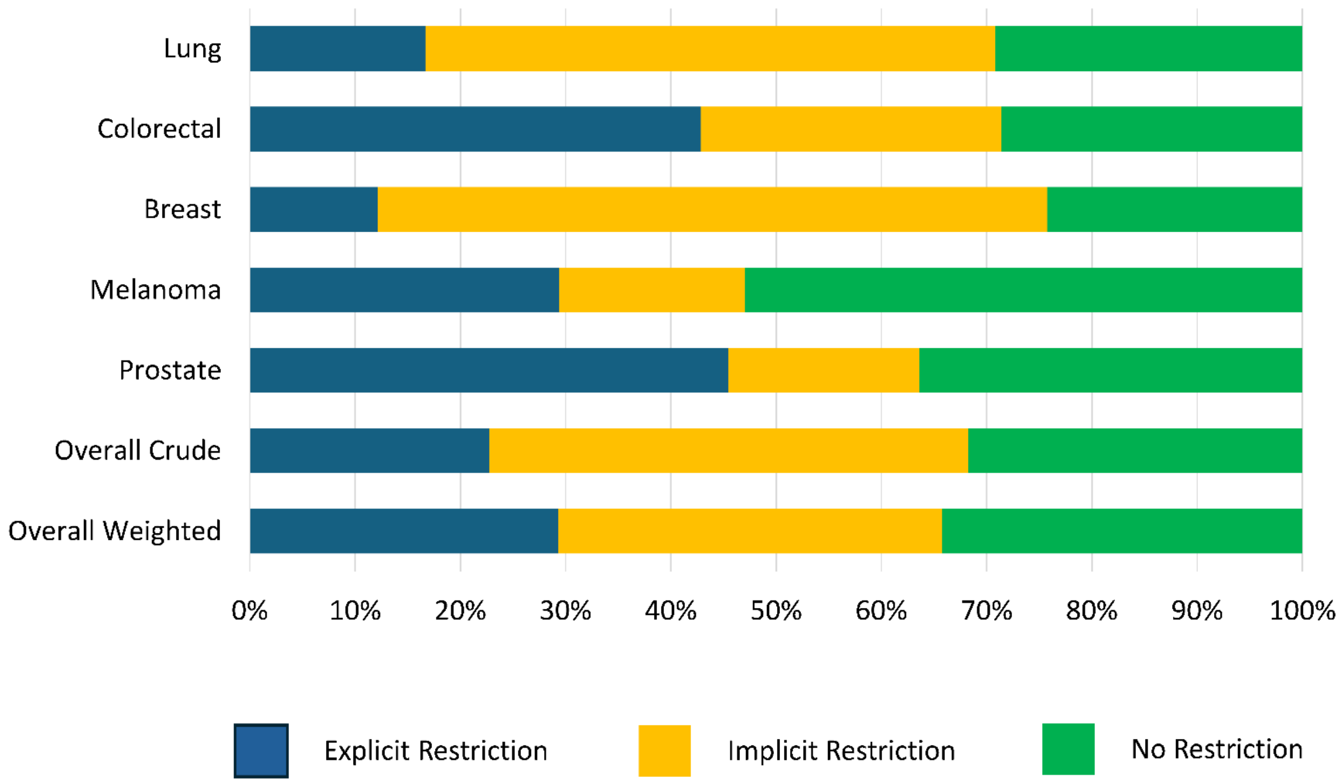

© 2024 Hibbs SP et al. *JAMA Network Open*.

eFigure 5. Proportions of SACT regimens with dose modifications that exclude patients for ANC values within the DANC reference range, by type of therapy. Regimens listed as chemotherapy are all those including cytotoxic chemotherapy (N=44). Those listed as targeted therapy regimens do not also include chemotherapy (N=11), and those listed as hormonal therapy do not also include chemotherapy or targeted therapy (N=16).

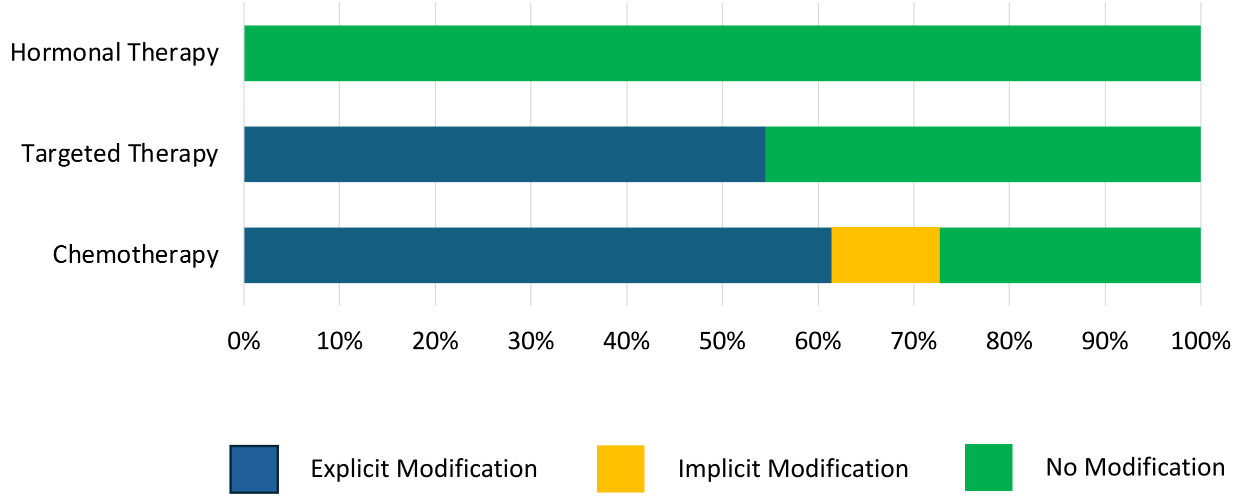

© 2024 Hibbs SP et al. *JAMA Network Open*.

eFigure 6: Chemotherapy and targeted therapy dose modifications that exclude patients for ANC values within the DANC reference range, by cancer type and type of restriction. The Overall Crude result is the sum of all regimens, and the Overall Weighted result weights equally by cancer type and then sums the results. Lung cancer (N=31), colorectal cancer (N=11), breast cancer (N=10), melanoma (N=3). Prostate cancer is not shown as all regimens identified for this cancer type were hormone-based.

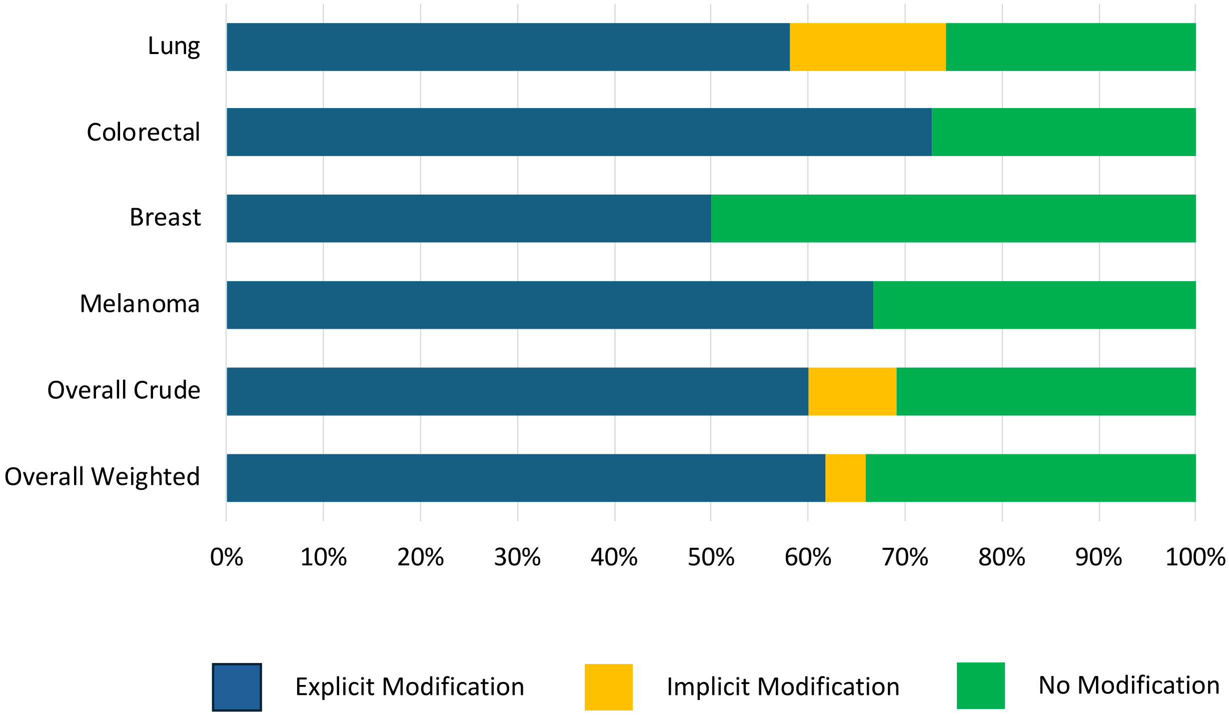

© 2024 Hibbs SP et al. *JAMA Network Open*.

eFigure 7: FDA label-based SACT dose modifications that exclude patients for ANC values within the DANC reference range, by cancer type and type of restriction. The Overall Crude result is the sum of all regimens, and the Overall Weighted result weights equally by cancer type and then sums the results. Lung cancer (N=31), colorectal cancer (N=11), breast cancer (N=14), melanoma (N=3), prostate (N=12).

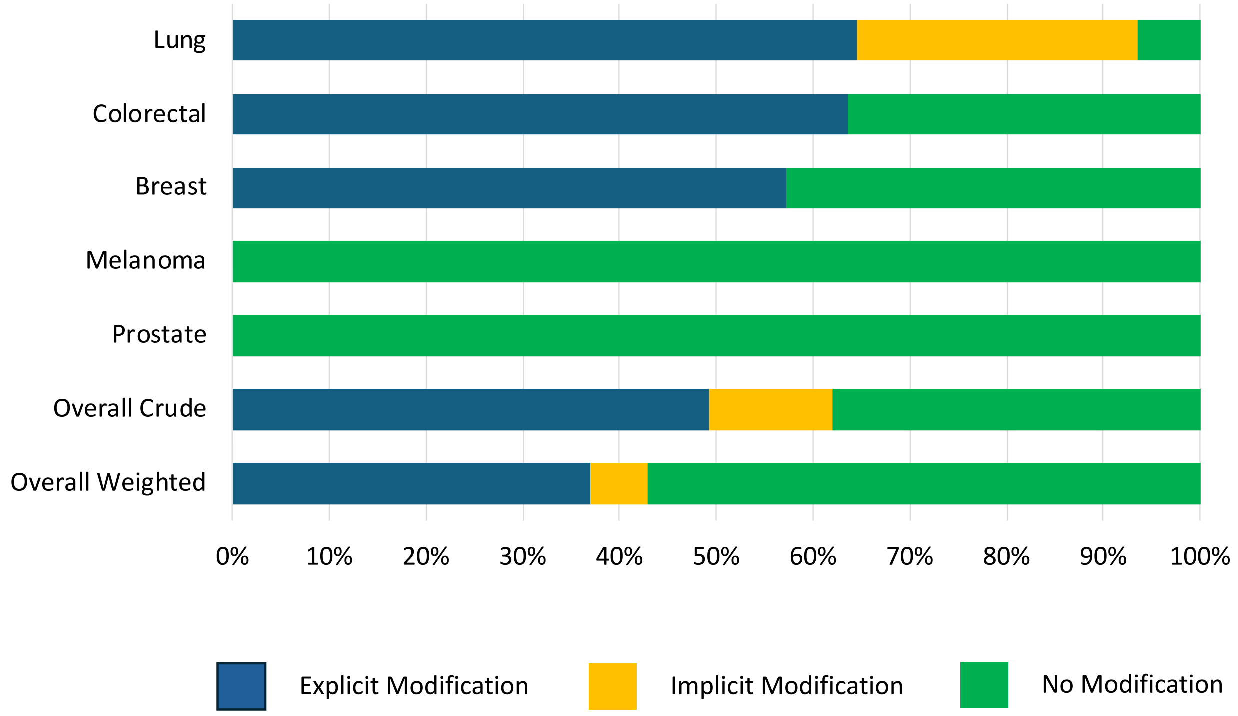

© 2024 Hibbs SP et al. *JAMA Network Open*.

References

1. Neuss MN, Gilmore TR, Belderson KM, et al. 2016 Updated American Society of Clinical Oncology/Oncology Nursing Society Chemotherapy Administration Safety Standards, Including Standards for Pediatric Oncology. *Journal of Oncology Practice*. 2016;12(12):1262-1271. doi:10.1200/jop.2016.017905

2. Shulman LN, Miller RS, Ambinder EP, Yu PP, Cox JV. Principles of Safe Practice Using an Oncology EHR System for Chemotherapy Ordering, Preparation, and Administration, Part 2 of 2. *Journal of Oncology Practice*. 2008;4(5):254-257. doi:10.1200/jop.0857501

© 2024 Hibbs SP et al. *JAMA Network Open*.
