# Supplementary material for: Cancer Trial Eligibility and Therapy Modifications for Individuals With Duffy Null–Associated Neutrophil Count
Source: JAMA Netw Open. 2024 Sep 11;7(9):e2432475. doi: 10.1001/jamanetworkopen.2024.32475 (PMC11391325; doi:10.1001/jamanetworkopen.2024.32475)
Supplement: Supplement 2. — Data Sharing Statement [file jamanetwopen-e2432475-s002.pdf]

Data Sharing Statement

Hibbs. Cancer Trial Eligibility and Therapy Modifications for Individuals With Duffy Null–Associated Neutrophil Count. *JAMA Netw Open*. Published September 11, 2024. doi:10.1001/jamanetworkopen.2024.32475

Data
Data available: No

Additional Information
Explanation for why data not available: The data presented are publicly available. A list of the studies and regimens included in the analysis, and data dictionaries of the data extracted related to these studies and regimens, are presented in the supplemental appendix. The extracted data are available upon reasonable request to the corresponding author.
